# Performance of Web tools for predicting changes in protein stability caused by mutations

**DOI:** 10.1186/s12859-021-04238-w

**Published:** 2021-07-05

**Authors:** Anna Marabotti, Eugenio Del Prete, Bernardina Scafuri, Angelo Facchiano

**Affiliations:** 1 Department of Chemistry and Biology “A. Zambelli”, University of Salerno, Fisciano, SA Italy; 2 CNR-IAC, National Research Council, Institute for Applied Mathematics “Mauro Picone”, Naples, Italy; 3 CNR-ISA, National Research Council, Institute of Food Science, Avellino, Italy

**Keywords:** Protein mutations, Protein stability, Rare diseases, Predictions, Statistical analysis

## Abstract

**Background:**

Despite decades on developing dedicated Web tools, it is still difficult to predict correctly the changes of the thermodynamic stability of proteins caused by mutations. Here, we assessed the reliability of five recently developed Web tools, in order to evaluate the progresses in the field.

**Results:**

The results show that, although there are improvements in the field, the assessed predictors are still far from ideal. Prevailing problems include the bias towards destabilizing mutations, and, in general, the results are unreliable when the mutation causes a ΔΔG within the interval ± 0.5 kcal/mol. We found that using several predictors and combining their results into a consensus is a rough, but effective way to increase reliability of the predictions.

**Conclusions:**

We suggest all developers to consider in their future tools the usage of balanced data sets for training of predictors, and all users to combine the results of multiple tools to increase the chances of having correct predictions about the effect of mutations on the thermodynamic stability of a protein.

**Supplementary Information:**

The online version contains supplementary material available at 10.1186/s12859-021-04238-w.

## Background

The impact of mutations on a protein can be different, depending on the nature of the protein and the type of mutation, but often they decrease the thermodynamic stability of the protein, thus promoting misfolding and/or aggregation [1]. Indeed, only a marginal gain in free energy is often associated with the correct three-dimensional structure of a protein compared to the misfolded states, and therefore this subtle balance can be altered even by a mutation that affects a single residue [2]. These phenomena are particularly evident in proteins associated with genetic disorders or other diseases that, through the mutation of a gene, determine the alteration of the corresponding protein, with loss of structure and protein function [3]. Thus, from a clinical perspective, the prediction of the effects of mutations on the stability of a protein is useful to identify therapeutic approaches for the treatment of diseases involving protein misfolding, such as the use of pharmacological chaperones [4]. These compounds either bind specifically to target proteins and stabilize their native conformation, or decrease the folding energy barriers, thereby promoting the transition of the misfolded forms into the native conformation [5].

The experimental assessment of the impact of a mutation on protein stability is not trivial, especially if hundreds of mutations are associated to a protein. Therefore, during the last three decades, dozens of computational methods have been developed to predict the alteration of the thermodynamic stability of a protein following mutations (for a recent and thorough review, see [6]). Their reliability has been questioned repeatedly by assessments performed in the past years [7–14], and, as a result, new methods have been developed more recently, which claim to overcome the limitations of the older methods [6]. Several new methods, which are often available online through Web servers, are based on different strategies, such as machine learning, which are supposed to produce more accurate results, but most have not been assessed by independent researchers.

In the past, we extensively used several of these methods to predict the impact of mutations on the stability of enzymes involved in the genetic disease galactosemia [15, 16]), and we noticed that often their predictions were not in agreement with each other. Moreover, comparing their predictions with the ΔΔG of the mutation obtained experimentally, we often found that the predictors were not fully reliable. In order to increase the reliability of our predictions, we performed an independent assessment of predictors that have emerged as promising or more reliable in the last few years. Here, we describe the problems that we dealt with, the solutions that we adopted, the results that we obtained, and the take-home messages learned from this study.

## Results

We evaluated five Web tools for the prediction of the effect of mutations on protein stability: DUET [17], INPS-3D [18], MAESTROweb [19], PoPMuSiC [12], DynaMut [20]. The most important features of each predictor are summarized in Table 1; for more details, the reader is referred to ref. [6] or to the original publications of the methods.

**Table 1:** Summary of the features of the predictors used in this study

Name of the predictor	Web server address	Input data	Type of approach	Dataset used for testing	References
PopMuSiC	http://www.dezyme.com/en/Software (accessible to registered users only; free of charge for academic users)	Protein structure	Linear combination of statistical potentials whose coefficients depend on the solvent accessibility of the mutated residue, correcting the bias toward destabilizing mutations imposing physical symmetries under inverse mutations	Direct dataset: S2648 dataset, made of 2648 different point-mutations (602 stabilizing and 2046 destabilizing) across 131 globular proteins with experimentally determined structures and impact on protein stability, extracted from ProTherm. Inverse dataset: constructed from all inverse mutations belonging to the direct dataset by modelling each inverse mutant with Modeller: 2648 mutations on 2648 different 3D structures	[12]
INPS-3D	https://inpsmd.biocomp.unibo.it/welcome/default/index	Protein sequence (INPS) or structure (INPS3D)	Support vector regression trained on descriptors encoding mutation type (in particular, substitution score, Hydrophobicity score, mutability index of native residue, molecular weights of native and mutant residues) and evolutionary information (INPS). Addition of structural features such as relative solvent accessibility of native residue and local energy difference calculated by a contact potential (INPS3D)	(i) S2648 dataset; (ii) a subset of S2648 used as blind test set comprising 351 variations in 60 proteins; (iii) a dataset of 42 variations within the DNA-binding domain of the tumor suppressor protein P53	[18]
DUET	http://biosig.unimelb.edu.au/duet/stability	Protein structure	Meta-predictor: consensus prediction of two complementary approaches developed by the same research group (mCSM and SDM) and obtained by combining the results using SVM	S2648 dataset. In particular: the training set comprises 2297 mutations randomly selected from the S2648 dataset; the test set is composed of the other 351 non-redundant mutations	[17]
DynaMut	http://biosig.unimelb.edu.au/dynamut/	Protein structure	Meta-predictor: consensus among different predictors (Bio3D, ENCoM and DUET). The first two predictors are based on normal mode analysis of the conformational variability, the last one is a consensus method based on two approaches calculating statistical potentials	Same as DUET	[20]
MAESTROweb	https://pbwww.che.sbg.ac.at/maestro/web	Protein structure	Predictor based on a multi-agent machine learning system estimation, which provides also high-throughput scanning for multi-point mutations and a specific mode for the prediction of stabilizing disulfide bonds	7 different datasets (4 for single point mutations—including S2648 dataset—for a total of 6688 mutations; 1 for multipoint mutations for a total of 479 mutations; 2 for mutations involving disulfide bonds, for a total of 90 disulfide bonds), extracted by ProTherm and by the datasets used to develop PoPMuSic, IMutant2.0 and AutoMute (see ref. 6 for the description of these tools)	[19]

We collected a benchmark dataset of monomeric and multimeric proteins (Additional files 13 and 14: S1 and S2 Files), with known values of ΔΔG associated with different mutations, starting from the dataset used by Vihinen and coworkers to develop their PON-tstab predictor [21], freely available in the VariBench benchmark database suite [22]. We predicted the change in stability of these mutant proteins and analysed the results of each predictor in order to determine their performance. As in this case the definition of both positive and negative predictions is arbitrary, we defined as positive predictions those mutations that caused a positive ΔΔG variation, and as negative predictions those mutations that caused a negative ΔΔG variation. We also separated those mutations that caused a ΔΔG variation ≤|0.5| kcal/mol from those mutation that caused a ΔΔG variation >|0.5| kcal/mol. In fact, the predictions for mutations with experimental ΔΔG variation in the range ± 0.5 kcal/mol are likely less reliable, because of their proximity to the experimental error [8]. The “Methods” section reports detailed information about the criteria used for creating the dataset and the statistical analysis used for the comparison.

### Analysis of the performance of stability predictors on the full dataset of monomeric proteins

Since most of the predictors of protein stability are set to analyse single-chain proteins with single mutations, we first assessed their predictions on the full dataset of monomeric proteins (see “Methods” section). The results are shown in Table 2. Considering the data predicted for mutations with a ΔΔG value outside the range of the experimental error (Table 2, upper part), we found PoPMuSiC and INPS-MD to be the predictors with the highest number of true negatives (i.e. destabilizing mutations that are correctly predicted as destabilizing) and false negatives (i.e. stabilizing mutations that are wrongly predicted as destabilizing). Conversely, we found DynaMut to be the predictor with the highest number of false positives (i.e. destabilizing mutations that are wrongly predicted as stabilizing) and true positives (i.e. stabilizing mutations that are correctly predicted as stabilizing), and with the lowest MCC value. However, the MCC values appeared to be relatively poor for all the predictors, considering that MCC = 1 indicates the best possible prediction and 0 a random prediction. As expected, the predictions made on mutations with a ΔΔG inside the range of the experimental error are poor (Table 2, lower part). In this case, PoPMuSiC gave the best result in terms of MCC, and INPS-MD and DynaMut gave the worst results. However, the MCC value appeared to be close to randomness for all predictors used on this subset of mutations.

**Table 2:** General results from the assessment on the full dataset of monomeric proteins

	PoPMuSiC	DynaMut	DUET	INPS-MD	MAESTROweb
*Values calculated for mutations causing a ΔΔG* > *|0.5|kcal/mol*					
True negative	424	270	413	422	399
False positive	19	173	30	21	44
True positive	44	80	64	49	62
False negative	58	22	38	53	40
Accuracy	0.86	0.64	0.88	0.86	0.85
True negative rate (specificity)	0.96	0.61	0.93	0.95	0.90
True positive rate (sensitivity)	0.43	0.78	0.63	0.48	0.61
Positive predictive value (precision)	0.70	0.32	0.68	0.70	0.58
Negative predictive value	0.88	0.92	0.92	0.89	0.91
MCC	0.47	0.31	0.58	0.50	0.50
*Values calculated for mutations causing a ΔΔG* ≤*|0.5|kcal/mol*					
True negative	101	58	87	93	75
False positive	17	60	31	25	43
True positive	27	50	33	25	43
False negative	52	29	46	54	36
Accuracy	0.65	0.55	0.61	0.60	0.60
True negative rate (specificity)	0.86	0.49	0.74	0.79	0.64
True positive rate (sensitivity)	0.34	0.63	0.42	0.32	0.54
Positive predictive value (precision)	0.61	0.45	0.52	0.50	0.50
Negative predictive value	0.66	0.67	0.65	0.63	0.68
MCC	0.23	0.12	0.16	0.12	0.18

True negative and true positive values have been considered as those predictions that correctly predicted a negative and a positive sign for destabilizing and stabilizing mutations, respectively

**Fig. 1:**
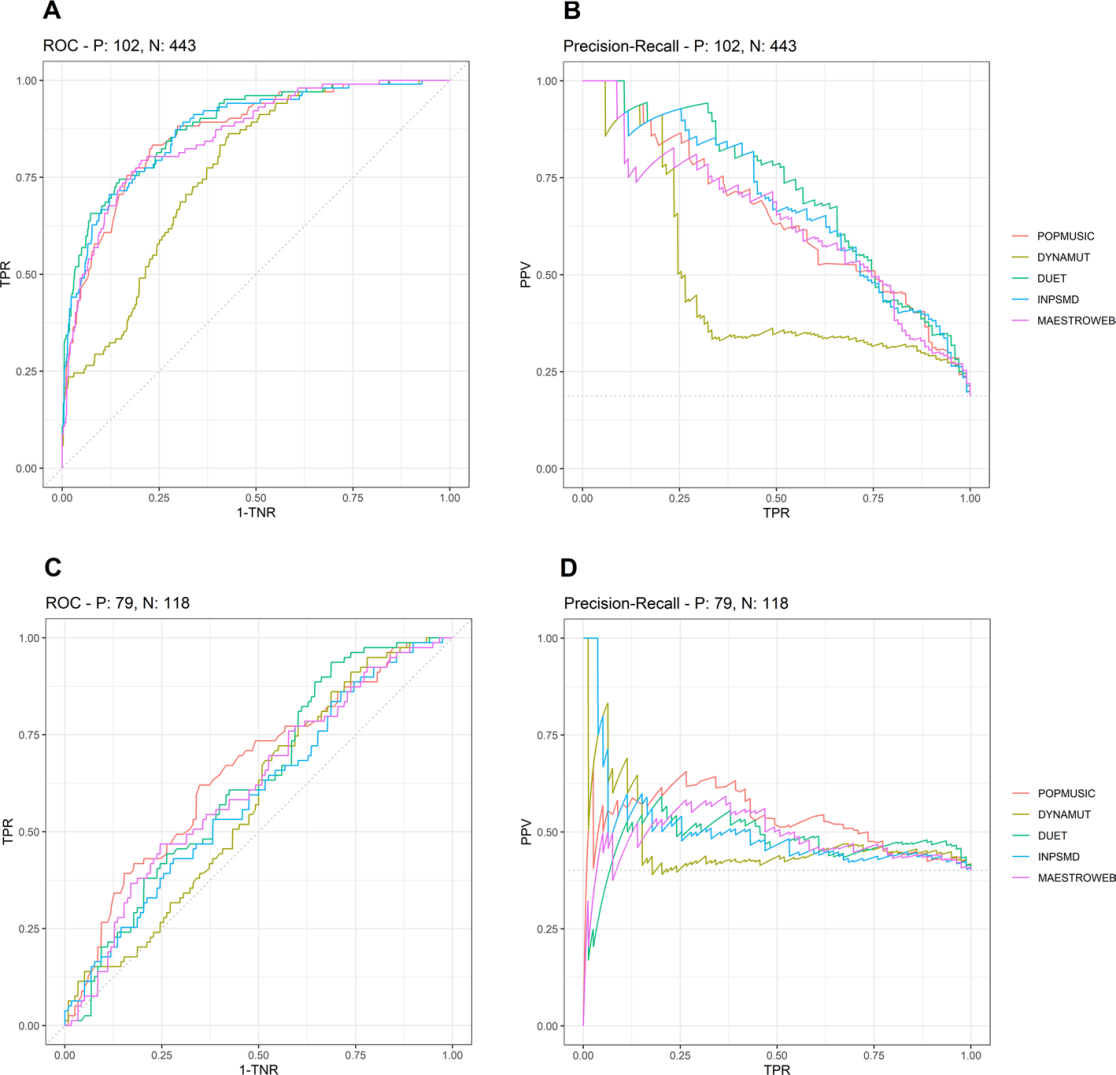
ROC and PRC for predictions made on the full dataset of monomeric proteins. Panels **a**, **b** show, respectively, the ROC and PRC for predictions made taking into account only those mutations with a ΔΔG value outside the range of the experimental error (number of positive elements is 102, number of negative elements is 443). Panels **c**, **d** show, respectively, the ROC and PRC for predictions made taking into account only those mutations with a ΔΔG value inside the range of the experimental error (number of positive elements is 79, of the negative elements is 118). TNR: True Negative Rate, TPR: True Positive Rate, PPV: Positive Predictive Value

Figure 1 shows the ROC (Receiver Operative Characteristic) and PRC (Precision-Recall Curve) calculated on the full dataset of monomeric proteins. Considering ROC, 4 out of 5 predictors (PoPMuSiC, DUET, INPS-MD, MAESTROweb) had similar performances, both in terms of true positive rate (TPR, or sensitivity) and true negative rate (TNR, or specificity), on predicting the effects of mutations on monomeric proteins when the ΔΔG variation was outside the experimental error (panel A), with a slightly lower performance of MAESTROweb and PoPMuSiC compared with DUET and INPS-MD. We found DynaMut to be the method nearest to the random dotted line, and its performance was worse than the other four methods analyzed. Considering PRC (panel B), we found that DUET had a slightly better performance in terms of positive predictive value (PPV), in the range [0.25, 0.63] of TPR. Again, DynaMut had the worst performance. The ROC for the monomeric subset inside the range of experimental error is shown in Fig. 1c. All of the five methods analyzed were very close to the random dotted line: as expected, these ΔΔG values could not be reliably predicted. The PRC for this subset is shown in Fig. 1d. Again, all five methods were very close to the random dotted line, with the rippled trends that do not show if a method is better than another.

This qualitative evaluation was confirmed by the values of AUC for both curves reported in Additional file 10: Table S1, from which it appears that all the methods but DynaMut have a difference of < 7% for the AUC related to both ROC and PRC, calculated for the monomeric subset outside the range of the experimental error, whereas DynaMut has a difference of around 16–24%. The AUC values for the curves related to predictions for ΔΔG inside the range of experimental error are lower and similar for all predictors (Additional file 10: Table S1).

Since in many cases the PDB file of a monomeric protein contains more than one chain, we performed a check to determine whether the positive or negative signs of the predictions made on each chain were in agreement. The results are shown in Additional file 11: Table S2. We found that PoPMuSiC was the only predictor with identical results (both in sign and in value) for all multiple chains, whereas the other predictors show different results among the chains, indicating that they are influenced by subtle differences in the structures of the different chains. Nevertheless, the sign of the predictions was usually the same for the different chains for all predictors, although DynaMut showed the lowest agreement among the chains, especially for monomeric proteins with three chains in their PDB file.

We also analysed the agreement among the different predictors for mutations on the monomeric dataset. The results are shown in Additional file 12: Table S3. As expected from the previous results, we found that DynaMut was the predictor that agreed least with the others, while the other predictors were in agreement for more than 80% of the predictions, with no significant differences among them.

In the database used for these analyses, the mutations to Alanine are about 5 times more frequent than the mutations to other types of residue (Additional file 14: File S2). We determined whether this unbalanced composition might represent a bias that affected the results of our assessment. Therefore, we created a subset of monomeric proteins by including all mutations except Alanine, and we recalculated ROC and PRC on this subset. The results (Additional file 1: Figure S1) showed that the ROC and PRC calculated for the subset of data without Alanine mutations were indistinguishable from the curves obtained for the entire dataset (Fig. 1a, b). In addition, the ROC and PRC calculated on the subset made up uniquely of monomeric proteins with mutations to Alanine are superimposable onto the general ROC and PRC (data not shown). Therefore, we concluded that the unbalanced composition of the dataset did not affect the results.

Another possible source of bias is the overrepresentation of mutations coming from a few specific proteins in the dataset. As shown in Additional file 14: File S2, three proteins each contributed more than 5% of the total mutations in the dataset of monomeric proteins. We therefore excluded these proteins and re-calculated the ROC and PRC curves. Results are reported in Additional file 2: Figure S2. Results showed that only the performance of DynaMut was remarkably improved in this subset.

In order to determine whether the size of the dataset affected the results, we performed a bootstrap analysis (sampling with replacement) by increasing the size of the subsets from 100 to 700 mutations, and measured how this affected the AUC values of the ROCs. These results are shown in Additional file 3: Figure S3, in which we reported the results for the largest and the smallest subsets. The increase in the dimensions of the dataset depicts narrower curves, but without a remarkable shift of their median value, indicating that even the smaller subset was of sufficient size to give reliable results, despite its spreader distribution. DynaMut remained the predictor with the lowest AUC value in both subsets, followed by MAESTROweb, while the performances of the other methods were similar.

### Analysis of the performance of stability predictors on a balanced dataset of monomeric proteins

Destabilizing mutations are overrepresented in the full dataset of monomeric proteins; thus, we calculated the performance of the predictors using a subset in which the number and the distribution of the ΔΔG of destabilizing and stabilizing mutations was well balanced (see Methods). The results are shown in Table 3. Using this dataset, we found that PoPMuSiC and INPS-MD had the highest number of true negative and false negative predictions, indicating that they are biased towards destabilizing mutations. A similar bias, although less evident, was also present for DUET and MAESTROweb. Conversely, DynaMut appeared to be more shifted towards stabilizing mutations, being the one with the highest number of true positive and false positive predictions.

**Table 3:** General results from the assessment on the balanced dataset of monomeric proteins

	PoPMuSiC	DynaMut	DUET	INPS-MD	MAESTROweb
*Values calculated for mutations causing a ΔΔG* *>|0.5|kcal/mol*					
True negative	94	52	89	95	86
False positive	8	50	13	7	16
True positive	44	80	64	49	62
False negative	58	22	38	53	40
Accuracy	0.68	0.65	0.75	0.71	0.73
True negative rate (specificity)	0.92	0.51	0.87	0.93	0.84
True positive rate (sensitivity)	0.43	0.78	0.63	0.48	0.61
Positive predictive value (precision)	0.85	0.62	0.83	0.88	0.79
Negative predictive value	0.62	0.70	0.70	0.64	0.68
MCC	0.40	0.31	0.52	0.46	0.46
*Values calculated for mutations causing a ΔΔG* *≤|0.5|kcal/mol*					
True negative	65	35	57	64	47
False positive	15	45	23	16	33
True positive	27	50	33	25	43
False negative	52	29	46	54	36
Accuracy	0.58	0.53	0.57	0.56	0.57
True negative rate (specificity)	0.81	0.44	0.71	0.80	0.59
True positive rate (sensitivity)	0.34	0.63	0.42	0.32	0.54
Positive predictive value (precision)	0.64	0.53	0.59	0.61	0.57
Negative predictive value	0.56	0.55	0.55	0.54	0.57
MCC	0.17	0.07	0.14	0.13	0.13

True negative and true positive values have been considered as those predictions that correctly predicted a negative and a positive sign for destabilizing and stabilizing mutations, respectively

**Fig. 2:**
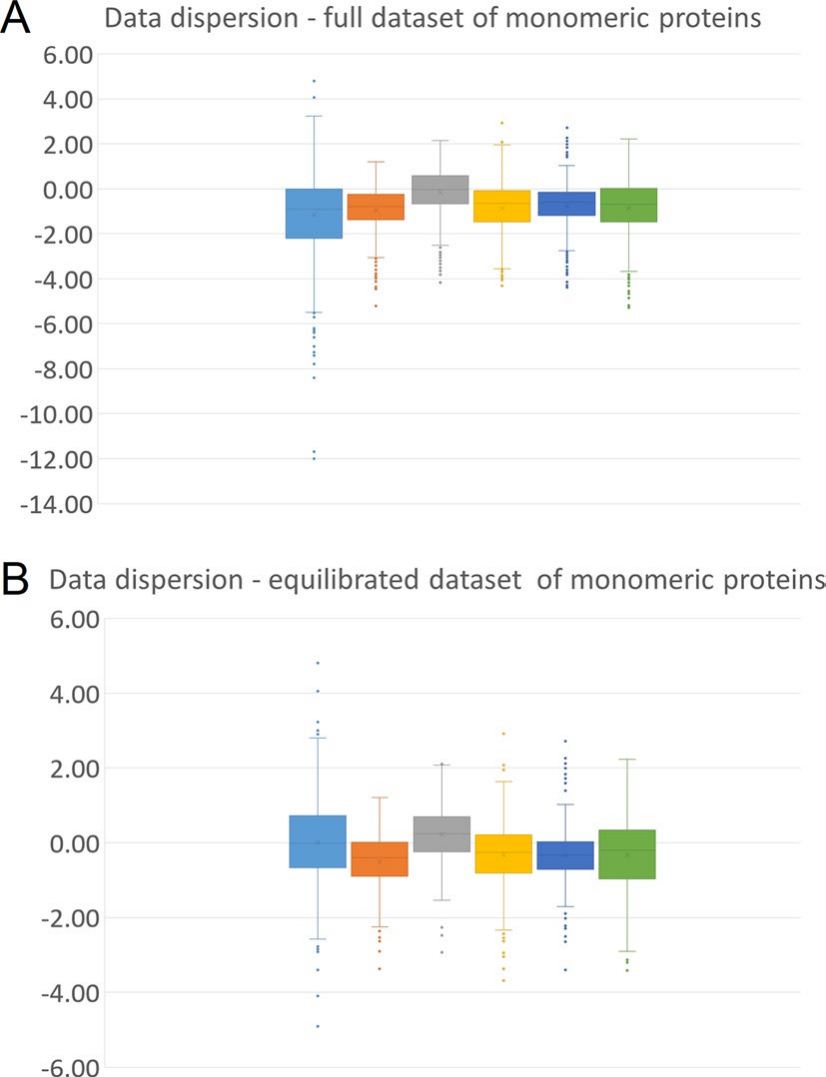
Box-plot of the distribution of the experimental and predicted ΔΔG values for the full dataset (**a**) and the balanced dataset (**b**) of mutations for the monomeric proteins. Cyan: distribution of the experimental ΔΔG; orange: predictions by PoPMuSiC; grey: predictions by DynaMut; yellow: predictions by DUET; blue: predictions by INPS-MD; green: predictions by MAESTROweb

Overall, we found that on the balanced dataset, considering the predictions for mutations with a ΔΔG value outside the range of the experimental error (Table 3, upper part), the performance of all of the predictors except DynaMut were less satisfactory compared to the previous unbalanced dataset. In particular, the accuracy decreased consistently for PoPMuSiC (from 0.86 to 0.68), DUET (from 0.88 to 0.75), INPS-MD (from 0.86 to 0.71), and MAESTROweb (from 0.85 to 0.73), while it was nearly unchanged for DynaMut (from 0.64 to 0.65). The true negative rate and the negative predictive value decreased for all methods, while the true positive rate was unchanged. On the contrary, the positive predictive value increased for all methods. Consequently, the MCC calculated for this dataset was lower for all of the predictors except DynaMut, which was essentially unaffected by the introduction of a more balanced dataset. This different trend of DynaMut is better shown in Fig. 2, where we compared the results of the different predictors versus the distribution of the real experimental values. In the full dataset, all predictors have a shift towards negative values, when compared to experimental values (Fig. 2a). Conversely, in the balanced dataset (Fig. 2b), all methods but DynaMut were still shifted to negative values. Finally, even in this balanced dataset, when the ΔΔG was inside the range of the experimental error (Table 3, lower part), the predictions of all of the Web tools were close to randomness, as indicated by MCC values close to 0.

### Analysis of performance of stability predictors on full and balanced datasets of multimeric proteins

The performance of the different predictors on the full dataset of multimeric proteins, considering only the mutations outside the range of the experimental error, is shown in Table 4, upper part, and Additional file 4: Figure S4. The performance of the predictors worsened slightly compared with the results obtained for the monomeric dataset, but there was no significant difference between the predictions made by MAESTROweb, which has a specific input option for multimeric proteins, and the other predictors. The analysis of the predictions based on the balanced dataset (Table 4, lower part, and Additional file 5: Figure S5) led to the same conclusions as those obtained from the monomeric dataset: all predictors except DynaMut were biased towards destabilizing mutations, while DynaMut still had the worst performance in terms of MCC values.

**Table 4:** General results from the assessment on the datasets of multimeric proteins

	PoPMuSiC	DynaMut	DUET	INPS-MD	MAESTROweb
*Values calculated for the full dataset of multimeric proteins*					
True negative	131	96	127	135	125
False positive	11	46	15	7	17
True positive	21	30	25	14	22
False negative	24	15	20	31	23
Accuracy	0.81	0.67	0.81	0.80	0.79
True negative rate (specificity)	0.92	0.68	0.89	0.95	0.88
True positive rate (sensitivity)	0.47	0.67	0.56	0.31	0.49
Positive predictive value (precision)	0.66	0.39	0.63	0.67	0.56
Negative predictive value	0.85	0.86	0.86	0.81	0.84
MCC	0.44	0.30	0.47	0.35	0.39
*Values calculated for the balanced dataset of multimeric proteins*					
True negative	36	21	31	35	31
False positive	2	17	7	3	7
True positive	21	30	25	14	22
False negative	24	15	20	31	23
Accuracy	0.69	0.61	0.67	0.59	0.64
True negative rate (specificity)	0.95	0.55	0.82	0.92	0.82
True positive rate (sensitivity)	0.47	0.67	0.56	0.31	0.49
Positive predictive value (precision)	0.91	0.64	0.78	0.82	0.76
Negative predictive value	0.60	0.58	0.61	0.53	0.57
MCC	0.46	0.22	0.38	0.29	0.32

True negative and true positive values have been considered as those predictions that correctly found a negative and a positive sign for destabilizing and stabilizing mutations, respectively. Data have been reported only for mutations causing a ΔΔG energy variation outside the range of the experimental error, in the full and the balanced datasets of multimeric proteins

### Analysis of performance of predictors on the full and balanced datasets of monomeric and multimeric proteins using a consensus of predictors

The MCC obtained for each predictor on our different subsets was still far from the ideal value of 1. In the past, we applied an empirical consensus method to the analysis of our Galactosemia Protein Dataset by attributing a classification to the mutants ("more stable"/"less stable") based on the consensus of at least two out of the three (2/3) selected methods [15]. In order to determine whether this empirical procedure improved the performance of the available predictors, we assessed the reliability of the consensus of the predictors for attributing an effect (destabilizing or stabilizing) on the mutants in all of our datasets. We analysed the results of the consensus both of three out of five (3/5) predictors, and of two out of three (2/3), checking in the latter case all possible combinations and selecting those that obtained the best MCC. The results are shown in Table 5. In general, we found that the MCC resulting from the consensus of the predictors was higher (both in full and in balanced datasets), compared to the MCC values calculated for every single predictor. In addition, the consensus of 3/5 predictors performed better in the full dataset of monomeric proteins, whereas the consensus of 2/3 predictors performed better in the balanced dataset of monomeric proteins and in the full dataset of multimeric proteins. In the case of the balanced dataset of multimeric proteins, both consensus performed as well as the method with the highest MCC value (PoPMuSiC).

**Table 5:** Consensus of the predictors

	Monomeric proteins		Multimeric proteins	
	3/5 methods	2/3 methods^a^	3/5 methods	2/3 methods^b, c^
*Values calculated for the full dataset*				
True negative	426	407	134	133
False positive	17	36	8	9
True positive	63	73	22	24
False negative	39	29	23	21
Accuracy	0.90	0.88	0.83	0.84
True negative rate (specificity)	0.96	0.92	0.94	0.94
True positive rate (sensitivity)	0.62	0.72	0.49	0.53
Positive predictive value (precision)	0.79	0.67	0.73	0.72
Negative predictive value	0.92	0.93	0.85	0.86
MCC	0.64	0.62	0.50	0.53
*Values calculated for the balanced dataset*				
True negative	92	87	36	36
False positive	10	15	2	2
True positive	63	73	21	21
False negative	39	29	24	24
Accuracy	0.76	0.78	0.69	0.69
True negative rate (specificity)	0.90	0.85	0.95	0.95
True positive rate (sensitivity)	0.62	0.72	0.47	0.47
Positive predictive value (precision)	0.86	0.83	0.91	0.91
Negative predictive value	0.70	0.75	0.60	0.60
MCC	0.54	0.57	0.46	0.46

True negative and true positive values have been considered as those predictions that correctly found a negative and a positive sign for destabilizing and stabilizing mutations, respectively. Data have been reported for mutations causing a ΔΔG energy variation outside the range of the experimental error, in the full and the balanced datasets of both monomeric and multimeric proteins

^a^Results obtained using DynaMut, DUET and INPS-MD

^b^ For the full dataset, results obtained using PoPMuSiC, DUET and MAESTROweb

^c^ For the balanced dataset, results obtained using PoPMuSiC, DUET and INPS-MD

## Discussion

It is not a trivial undertaking to measure the effect of point mutations on the thermodynamic stability of a protein on a large scale. Many computational methods have been developed to predict the ΔΔG associated with protein mutations, and most of them are freely available via Web interfaces and provide their predictions quickly (from a few minutes to a few hours) [6]; thus, they are easy to use for "naïve users" in the field of bioinformatics. Nevertheless, several evaluations made in the last few years have questioned their reliability [7–14]. For this reason, we decided to perform a new assessment, starting from a “naïve user” perspective, to the prediction of whether a particular mutation, or set of mutations, might destabilize a particular protein. From our recent overview of the predictors that are currently available [6], we selected five Web server tools with the aim of testing structure-based predictors, representative of the different approaches developed to cope with the abovementioned issue, freely accessible to the users, and which allow calculating many mutations in a reasonable time. Moreover, considering that several assessments in the past have raised the question of whether many predictors are biased towards destabilizing mutations, we specifically addressed this issue by creating a subset of balanced mutations, upon which we evaluated the performance of the different predictors. Results on the balanced dataset indicate worse performances of the predictors, suggesting that the abundancy of destabilizing mutations in unbalanced datasets might mask the bias of the predictors towards destabilizing mutations.

Comparing our results with those obtained 10 years ago by Khan and Vihinen [8] in an assessment that used the same metrics, it is evident that the performance of the most recent predictors is generally improved, but the road towards the full reliability of their predictions is still long and winding. Indeed, although the accuracy and true negative rate are greatly improved, the true positive rate remains unchanged with respect to the older statistics. The MCC calculated for most of the methods available 10 years ago was close to 0, indicating that most of the predictions were close to randomness, and the best performing method showed an MCC of 0.27 [8]. In this study, the highest MCC value obtained by the best performing method is 0.58, twice that value, but it is still far from the ideal value of 1.

The results we obtained on the balanced dataset (Table 3) show that destabilizing mutations are better predicted than stabilizing ones by all predictors except DynaMut. Interestingly, we found that this tool had the highest number of true positives predictions (i.e. stabilizing mutations correctly predicted to have a stabilizing effect) in all the tested conditions. However, it was also the one with the highest number of false positive predictions (i.e. destabilizing mutations wrongly predicted to have a stabilizing effect) and this is the reason for its poor results in terms of MCC in all tested datasets.

Most of available tools are not able to predict the effect of concurrent mutations on the different chains of a multimeric protein. Therefore, the predictions have to be made for a single subunit at a time. Thus, it is difficult to derive an overall result for the whole protein, as the developers do not supply any indication on how to use their predictors in this case. To address this issue, we tested whether the prediction of the ΔΔG variation on the whole protein obtained with MAESTROweb (the only tool able to manage multimeric proteins) was comparable with the sum of the ΔΔG values of the single subunits predicted by the same tool. We calculated a correlation coefficient of 0.728, therefore we deduced that summing up the predictions of the ΔΔG variation of the single subunits was an acceptable approximation to evaluate the effect of concurrent mutations on the stability of a multimeric protein. The results obtained on multimeric proteins for all predictors (Table 4) show that MAESTROweb does not outperform the other tools, despite it has been tailored to manage also multimeric proteins.

When the experimental ΔΔG value of a mutation is close to the experimental error (± 0.5 kcal/mol) [8], all the tools return essentially random predictions for this mutation. It is worth noting that the predictors currently associate a binary output (destabilizing/stabilizing) to the predicted ΔΔG value, with two negative consequences: 1. in the "twilight zone" of the experimental error, the risk of having an unreliable prediction is very high; 2. the naïve user is misled by the interpretation of the real effect of the mutation. Indeed, a ΔΔG value of − 0.01 kcal/mol measured for a mutation means that the stability of the protein is unchanged, but these predictors would classify such a result as a "destabilizing effect". Therefore, we advise all users to look with caution at the predictions for which the ΔΔG values fall within the range of the experimental error, and we suggest all developers to define the effect of a mutation on the stability of the protein as "uncertain" when its predicted ΔΔG falls within the range ± 0.5 kcal/mol.

Finally, using the results of single predictors to perform an in-house consensus procedure, we obtained predictions with an increased reliability compared with the single best performing method. This solution provides a sort of compensation in the predictions, which allows overcoming the problems of every single method. Moreover, even the single best performing method in our assessment in terms of MCC value (DUET) is, in its turn, a consensus method. Therefore, based on our experience, we advise all users to be unsatisfied with a single prediction, and to combine the results of multiple tools to increase the chances of having a correct prediction about the effect of a mutation on the thermodynamic stability of a protein.

## Conclusions

The web tools used in this work perform surely better than the predictors assessed about 10 years ago, although improvements are still needed. As take home messages, this work suggests on an hand to developers to consider the needs of training their predictors on balanced datasets and evaluating carefully the cases with very low impact on the thermostability, and on the other hand to users to compare results from different predictors to obtain more reliable evaluations.

## Methods

### Web tools assessed for the prediction of protein stability

After a careful evaluation of the different approaches available to predict the impact of the mutations on the thermodynamic stability of a protein [6], we selected the following predictors to perform our assessment: DUET [17] (http://biosig.unimelb.edu.au/duet/), a web tool combining two other complementary predictors (named SDM and mCSM), previously developed by the same research group, in a consensus predictor; INPS-3D [18] (https://inpsmd.biocomp.unibo.it/inpsSuite/default/index3D), a machine-learning method tailored to address the problem of anti-symmetric property; MAESTROweb [19] (https://pbwww.che.sbg.ac.at/maestro/web), the only Web server able to manage both multimeric proteins and compound heterozygous multiple mutations; PoPMuSiC (https://soft.dezyme.com/query/create/pop; accessible to registered users only, freely available for academic users), a method formed by a linear combination of statistical potentials, the coefficients of which depend on solvent accessibility of the mutated residue. The new version of this predictor, developed by Pucci and coworkers in 2018 and called PoPMuSiCSym [12] has been tailored to correct the bias toward destabilizing mutations by imposing physical symmetries under inverse mutations; however, the version of the predictor executed by the Web server is called “PoPMuSiC”. Finally, DynaMut [20] (http://biosig.unimelb.edu.au/dynamut/) is one of the most recent Web servers developed, based on a quite original approach in which Normal Mode analysis is performed to take into account the contribution of protein flexibility.

These predictors were selected because they are freely available online and they are representative of the different approaches developed during the last few years [6]. Moreover, PoPMuSiC was selected because it appears to be the best tool available from the last extensive comparison made by Pucci et al. [12], and DUET because it is among the most popular stability predictor servers (as per Google Scholar citations). The other three predictors are more recent and were not previously assessed by researchers different from their developers. In particular, INPS-MD was selected because its authors stated that they carefully designed the training set for its development, to overcome the bias for destabilizing mutations [18]. Finally, DynaMut was selected because it is very popular (as per Google Scholar citations), and MAESTROweb for its unique ability to manage multimeric proteins.

In order to predict the change in protein stability, all of these tools require: (a) the structure of the wild-type protein in PDB format; (b) the specification of the missense mutation that the user wishes to study; (c) the protein chain involved in the mutation. As output, each tool provides the predicted ∆∆G value of the mutant compared with the wild type protein, and a binary classification of the mutation (either stabilizing or destabilizing) based on the sign of the predicted ∆∆G value. A negative sign for this value is associated with destabilizing mutations for DynaMut, DUET and INPS-MD, whereas PoPMuSiC and MAESTROweb associate a negative sign to stabilizing mutations. In order to standardize this binary output and make the statistical analyses easier, we inverted the sign of the output of PoPMuSiC and MAESTROweb in our statistical evaluations.

We defined arbitrarily as positive predictions those mutations associated to a positive ∆∆G sign and as negative predictions those mutations associated to a negative ∆∆G sign. As this assumption can affect non-symmetric evaluations such as ROC and PRC curves, we report under Additional files 6 to 9: Figures S6 to S9 the results of ROC and PRC curves obtained when the opposite choice is applied, i.e., positive predictions are associated to negative ΔΔG predictions.

### Dataset used for the assessment

To create the reference dataset for our assessment, we started from the benchmark dataset of proteins used by Vihinen and coworkers to develop their PON-tstab predictor [21], freely available at the VariBench database suite [22] (http://structure.bmc.lu.se/VariBench/stability.php; Dataset 5). This dataset, extracted from ProTherm [23], is probably the best curated resource of its kind currently available, but it still shows limitations due to its redundancy in terms of protein families, to its compositional bias in terms of type of mutations included, to an imbalance between destabilizing and stabilizing mutations and to the quality of the reference structures indicated [6]. To overcome these limitations, we created a “reference standard dataset”, extracting only high-quality reference proteins from VariBench. At the purpose, we first discarded: 130 mutations for which a reference structure was not cited in ProTherm; three proteins for which a discrepancy was present between the mutations reported in the sequence and the structure (e.g. mutation in the sequence not corresponding to the position of the mutation in the structure); several proteins for which the reference structure was obtained by NMR; one protein in which only the Cα trace was present in the reference structure; one protein in which 19 mutations involve a residue included in a missing segment in the structure. We also discarded several proteins for which the quality of the structure was judged unsatisfactory, when evaluated in accordance with the following criteria: resolution, R free/R value difference, Ramachandran outliers, sidechain outliers, normalized real-space R-value (RSRZ) outliers, geometric quality of the chain [24]. Finally, we discarded several proteins in which the reference (wild type) structure contained some mutations. The group of mutations of phage T4 lysozyme represented a particular case. The two reference structures for this protein indicated in the original dataset were 2LZM [25] and 1L63 [26]. Both of which are considered as wild type proteins, but 1L63 is the structure of the so-called “pseudo wild type “ or “cysteine-free lysozyme”, in which Cysteine 54 and Cysteine 97 were replaced by Threonine and Alanine, respectively, in order to disrupt the existing disulfide bridge between these two residues [27]. Comparing the mutations reported in the PON-tstab dataset and their reference in the literature, we kept in our “reference” dataset only those mutations for which we identified the reference lysozyme used for the experimental determination of ΔΔG.

Our final “reference standard” dataset for benchmarking includes 48 proteins, of which 10 are homodimeric, 1 is homotetrameric and the others are monomeric, according to the information of their biological assembly, derived from the indications in RCSB PDB database [28], from the literature associated to each structure and from visual inspection to solve ambiguities. In some cases, the multimeric proteins show only one chain in the PDB file: in these cases, we reconstructed the biological assembly by means of MakeMultimer.py tool, available at http://watcut.uwaterloo.ca/tools/makemultimer/. On the other hand, many monomeric proteins show more than one (identical) chain in their PDB file: in these cases, we decided to submit each chain independently for the prediction. For the monomeric proteins with more than one chain in their PDB files, we calculated the predictions separately for each chain, and then we averaged the value of ΔΔG for each mutation and predictor. For multimeric proteins, only MAESTROweb allows to calculate directly the effect of mutations on a protein composed by more than one chain. Therefore, we performed the prediction by using the appropriate settings for multimeric prediction available on the Web server, whereas for the other predictors we performed the predictions separately for each chain, and then summed the value of ΔΔG for each mutation and predictor.

Our reference dataset contains the effects of 1024 mutations introduced in the 48 proteins selected. We considered 585 mutations as “destabilizing” (ΔΔG < -0.5 kcal/mol), 168 as “slightly destabilizing” (− 0.5 ≤ ΔΔG < 0 kcal/mol), 103 as “slightly stabilizing” (0 < ΔΔG ≤ 0.5 kcal/mol), 147 as “stabilizing” (ΔΔG > 0.5 kcal/mol), and we also included in the dataset 21 mutations with ΔΔG = 0. Mutations affecting monomeric proteins are 759, mutations affecting multimeric proteins are 265.

From these data, it appears that the distribution of mutations with ΔΔG < 0 and ΔΔG > 0 is unbalanced; in particular, mutations with ΔΔG < 0 are three times those with ΔΔG > 0. Therefore, we derived two subsets of mutations, one for monomeric and one for multimeric proteins, well balanced for the distribution of ΔΔG, by keeping all stabilizing mutations and selecting an equal number of destabilizing mutations. The criteria to balance the dataset were: (1) ΔΔG distribution: we divided the dataset into ΔΔG ranges (in absolute value), and selected for each ΔΔG range an equal number of destabilizing and stabilizing mutations; (2) mutant protein: for each ΔΔG range, we selected destabilizing mutations coming from the same protein, or at least coming from proteins from the same structural family, as identified by the CATH code [29]; (3) type of mutation: for each ΔΔG range, we selected a balanced number of mutations of the same type (e.g. a mutation replacing a hydrophobic residue by a negatively charged one). These two subsets were used to assess the bias of the predictors towards destabilizing mutations.

The original dataset, the reference dataset and the two balanced subsets are provided as Additional file 13: File S1. A detailed analysis of our reference dataset composition in terms of type of proteins, type of mutations, distribution according to experimental ΔΔG, residue distribution, contributions of different proteins, structural features is reported in Additional file 14: File S2.

As reported in Table 1, the datasets used for training all the predictors include, or are derived, from the so-called "S2648 dataset", made of 2648 different point-mutations (602 stabilizing and 2046 destabilizing) across 131 globular proteins with experimentally determined structures and impact on protein stability, extracted from ProTherm (freely available at the VariBench database suite [22] (http://structure.bmc.lu.se/VariBench/stability.php; Dataset 10). This dataset was originally developed in 2009 to train a previous version of PoPMuSiC and therefore is less curated than the one we used to assess the performances of the predictors. Our dataset includes 28 proteins (out of 48) and 641 mutations (out of 1024) in common with the S2648 dataset (Additional file 14: File 2 details also this information). However, as we pointed out previously, in our dataset all chains included in the PDB files of the 28 proteins were taken into account, whereas only the first chain of the PDB file was taken as reference in S2648 dataset. Moreover, in our dataset multimeric proteins were considered in their quaternary assembly, whereas in the S2648 dataset all proteins were treated as monomeric. For these reasons, our dataset cannot be considered truly overlapping the S2648 dataset.

### Criteria and statistical analysis used to perform the assessment

As our main goal was to assess if predictors correctly identify those mutations that destabilize or stabilize the protein of interest, we evaluated whether the sign of the ΔΔG predicted by the different tools was in agreement with the sign of the experimental measure associated to the same mutation.

In our assessment, we evaluated the performance of the predictors separately for those mutations with a ΔΔG value comparable to the experimental error made to measure ΔΔG variation (between − 0.5 and 0.5 kcal/mol [8]), and for mutations with a ΔΔG value falling outside this range.

To evaluate the consensus as a possible method to improve the performances of the predictors, we tested the agreement of 3 out of 5 predictors, and 2 out of 3 by testing all possible combinations of 3 predictors.

The statistical measures used to assess the performances of the predictors were: accuracy (closeness of the measurements to a specific value), sensitivity (recall, or true positive rate), specificity (selectivity, or true negative rate), precision (positive predictive value), negative predictive value, and Matthew’s Correlation Coefficient (MCC). Sensitivity and specificity depict the Receiver Operating Characteristic (ROC) curve, whereas precision and sensitivity depict the Precision-Recall curve (PRC) most suitable for unbalanced datasets. The Area Under the Curve (AUC) for both curves is the performance value used for the comparison of the various prediction methods [30]. All the analyses on the prediction performances for the five methods were carried out in R language, with the confusion matrix values calculated with the **precrec** package [31], and the curves graphed with the help of the **ggplot2** package [32].

In order to evaluate the robustness of our results compared with the size of our dataset, we evaluated the performance of the monomeric dataset outside the range of the experimental error, in order to determine: (a) whether the size of our dataset is sufficient to reach a stable value of AUC (ROC) for all five methods of prediction; (b) whether it was possible to reach similar AUC values with a smaller dataset, which can be used as novel benchmark. For this purpose, we proceeded by following the technique of bootstrap:

1. The dataset was divided into 100 subsets (iterations) by resampling with replacement, with different dimensions, from 100 to 700 mutations in steps of 100, with a total of 700 subsets;
2. The procedure for the extraction of the AUC values was applied on all the subsets, with the storage of the results;
3. The density functions for the AUC values were plotted, separated for subset size and prediction method.

## Supplementary Information

**Additional file 1: Fig. S1**. ROC and PRC for predictions made on the full dataset of monomeric proteins deprived of mutations to Ala. Panels (A) and (B) show the ROC and PRC, respectively, for predictions made taking into account only those mutations outside the range of experimental error (ΔΔG > |0.5| kcal/mol) (number of positive elements is 88, number of negative elements is 308). TNR: True Negative Rate, TPR: True Positive Rate, PPV: Positive Predictive Value.

**Additional file 2: Fig. S2**. ROC and PRC for predictions made on the dataset of monomeric proteins deprived of mutations coming from the 3 most represented proteins. Panels (A) and (B) show the ROC and PRC, respectively, for predictions made taking into account only those mutations outside the range of experimental error (ΔΔG > |0.5| kcal/mol) (number of positive elements is 81, number of negative elements is 260). TNR: True Negative Rate, TPR: True Positive Rate, PPV: Positive Predictive Value.

**Additional file 3: Fig. S3**. AUC curve density obtained taking into account only those mutations outside the range of the experimental error, with a dataset of 100 (panel A) and 700 mutations (panel B), randomly extracted from the full dataset of monomeric proteins.

**Additional file 4: Fig. S4**. ROC and PRC for predictions made on the full dataset of multimeric proteins. Panels (A) and (B) show the ROC and PRC, respectively, for predictions made on multimeric proteins, taking into account only those mutations outside the range of the experimental error (number of positive elements is 45, number of negative elements is 142). TNR: True Negative Rate, TPR: True Positive Rate, PPV: Positive Predictive Value.

**Additional file 5: Fig. S5**. Box-plot of the distribution of the experimental and predicted ΔΔG values for the full (panel A) and balanced (panel B) datasets of mutations for the multimeric proteins. Cyan: distribution of the experimental ΔΔG; orange: distribution of ΔΔG predictions for PoPMuSiC; grey: distribution of ΔΔG predictions for DynaMut; yellow: distribution of ΔΔG predictions for DUET; blue: distribution of ΔΔG predictions for INPS-MD; green: distribution of ΔΔG predictions for MAESTROweb.

**Additional file 6: Fig. S6**. ROC and PRC curves obtained as for Fig. 1 by considering negative ΔΔG predictions as positives.

**Additional file 7: Fig. S7**. ROC and PRC curves obtained as for Additional file 1: Figure S1 by considering negative ΔΔG predictions as positives.

**Additional file 8: Fig. S8**. ROC and PRC curves obtained as for Additional file 2: Figure S2 by considering negative ΔΔG predictions as positives.

**Additional file 9: Fig. S9**. ROC and PRC curves obtained as for Additional file 4: Figure S4 by considering negative ΔΔG predictions as positives.

**Additional file 10: Table S1**. Quantitative values of AUC calculated for the full dataset of monomeric structures.

**Additional file 11: Table S2**. Consensus among the sign of predictions made on monomeric proteins with more than one chain in their PDB file.

**Additional file 12: Table S3**. Consensus among the sign of predictions made on monomeric proteins among the different predictors. Values are expressed as percentage.

**Additional file 13: File S1**. Reference monomeric and multimeric datasets. Data sheets report the original PON-tstab dataset, the selection of proteins for the full dataset, the full datasets (monomeric and multimeric proteins) with predictions, the balanced datasets.

**Additional file 14: File S2**. Analysis of the composition of the full datasets

## Data Availability

Reference monomeric and multimeric datasets used in this work are available as Additional file 13: File 1. Predictors used are available at the following URLs: http://www.dezyme.com/en/Software (PopMuSiC); https://inpsmd.biocomp.unibo.it/welcome/default/index (INPS-3D); http://biosig.unimelb.edu.au/duet/stability (DUET); http://biosig.unimelb.edu.au/dynamut/ (DynaMut); https://pbwww.che.sbg.ac.at/maestro/web (MAESTROweb).

## Abbreviations

AUC: Area Under the Curve
MCC: Matthew’s Correlation Coefficient
NMR: Nuclear Magnetic Resonance
PDB: Protein Data Bank
PRC: Precision-Recall Curve
ROC: Receiver Operative Characteristic
TNR: True negative rate, or specificity
TPR: True positive rate, or sensitivity
