# Supplementary figures and images for: Performance of Web tools for predicting changes in protein stability caused by mutations
Source: BMC Bioinformatics. 2021 Jul 5;22(Suppl 7):345. doi: 10.1186/s12859-021-04238-w (PMC8256537; doi:10.1186/s12859-021-04238-w)

**A B**


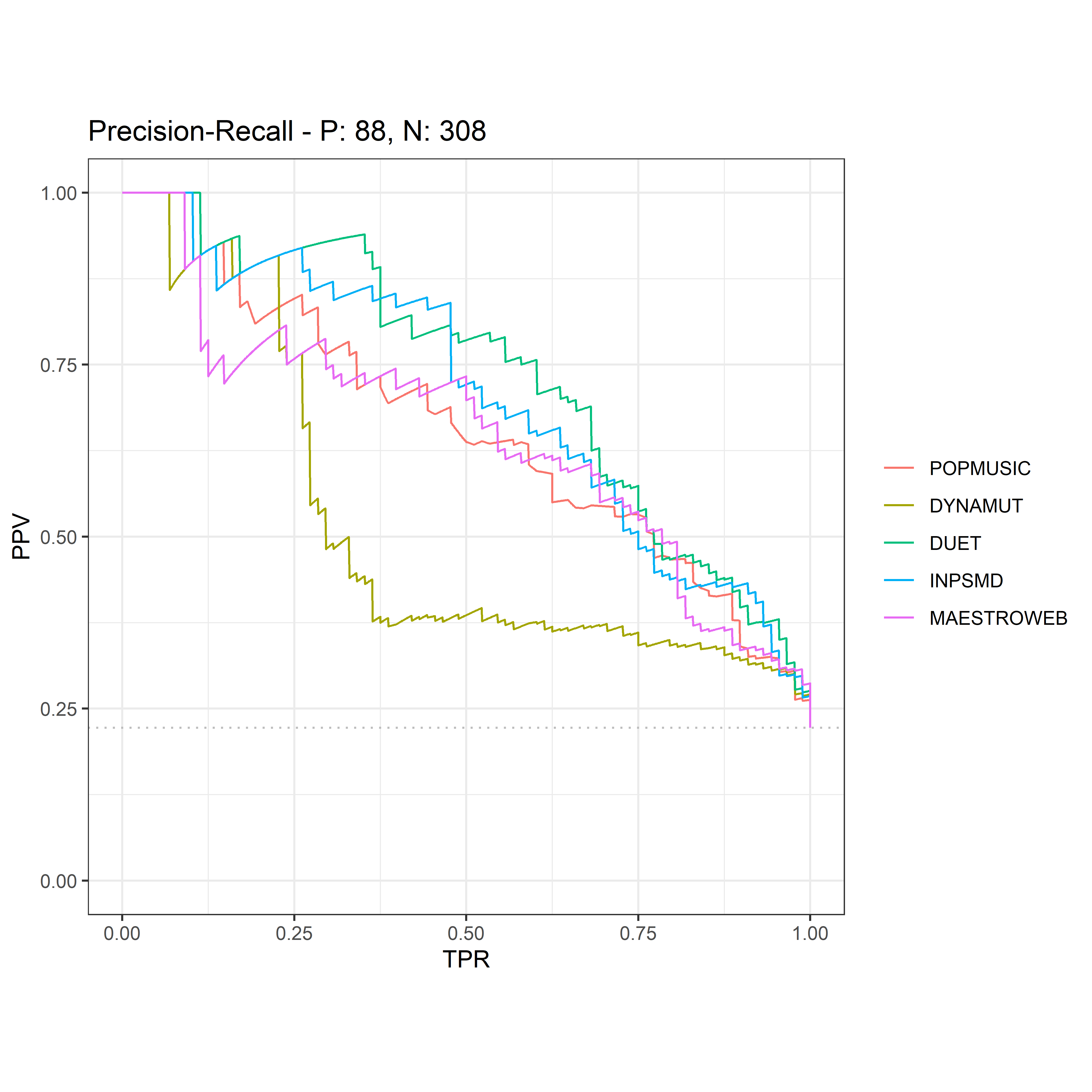

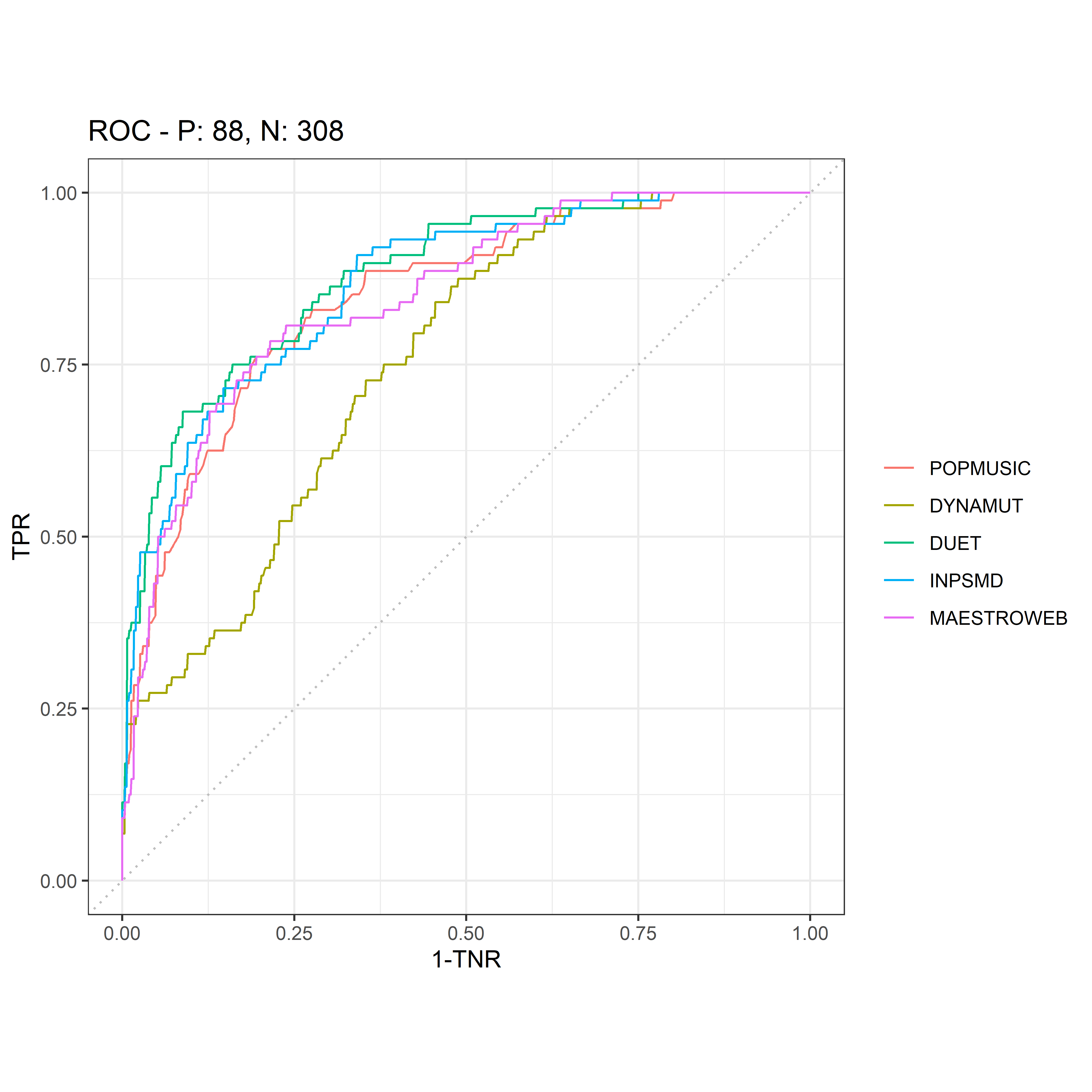

Supplement: Supplementary file 1 — Additional file 1: Fig. S1. ROC and PRC for predictions made on the full dataset of monomeric proteins deprived of mutations to Ala. Panels (A) and (B) show the ROC and PRC, respectively, for predictions made taking into account only those mutations outside the range of experimental error (ΔΔG > |0.5| kcal/mol) (number of positive elements is 88, number of negative elements is 308). TNR: True Negative Rate, TPR: True Positive Rate, PPV: Positive Predictive Value. [file 12859_2021_4238_MOESM1_ESM.docx]

**A B**

**
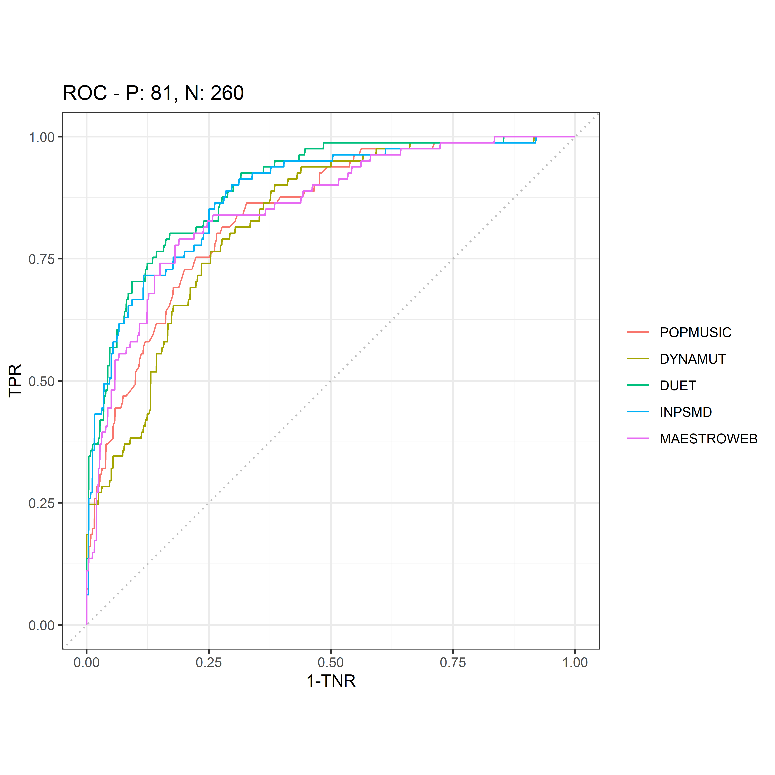
**


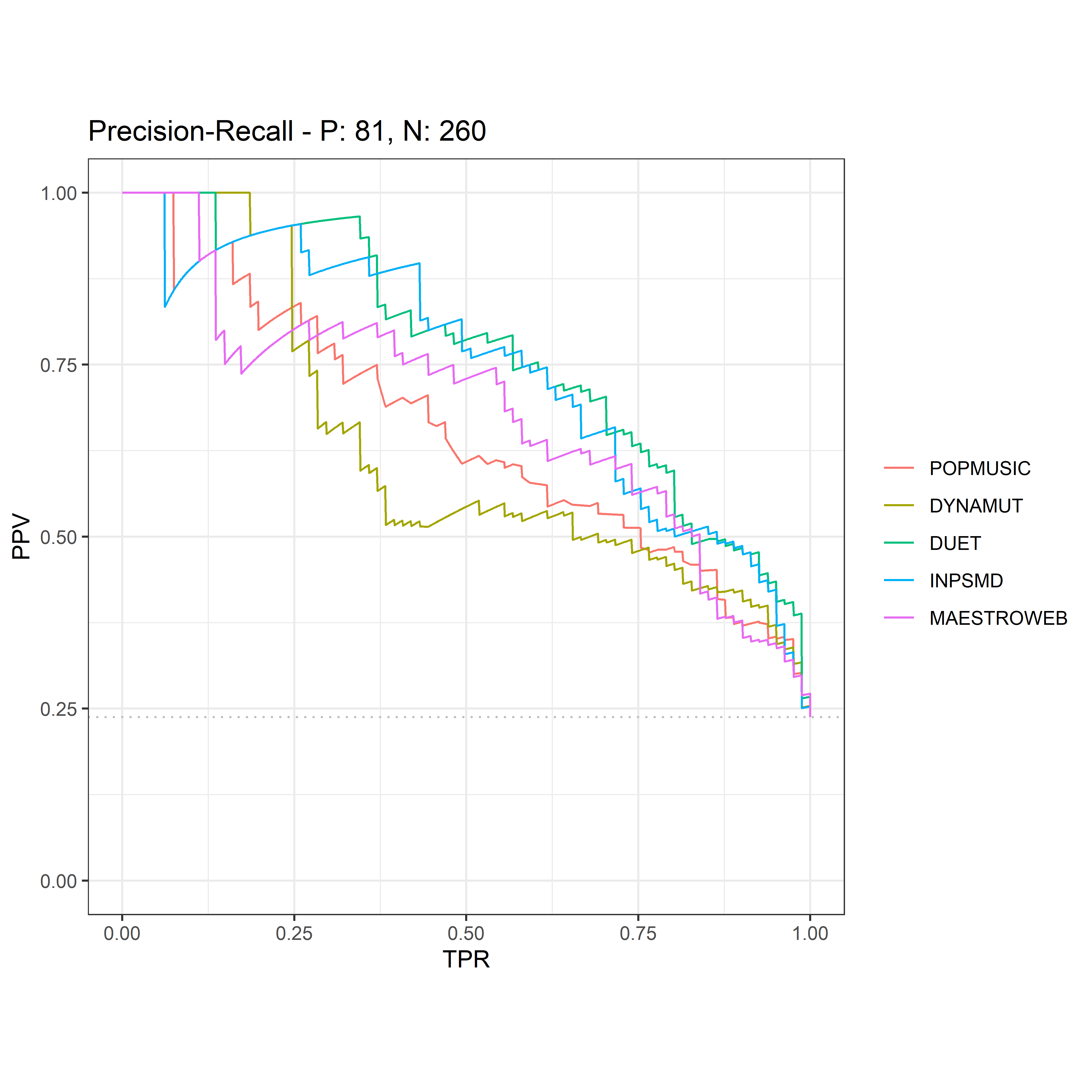

Supplement: Supplementary file 2 — Additional file 2: Fig. S2. ROC and PRC for predictions made on the dataset of monomeric proteins deprived of mutations coming from the 3 most represented proteins. Panels (A) and (B) show the ROC and PRC, respectively, for predictions made taking into account only those mutations outside the range of experimental error (ΔΔG > |0.5| kcal/mol) (number of positive elements is 81, number of negative elements is 260). TNR: True Negative Rate, TPR: True Positive Rate, PPV: Positive Predictive Value. [file 12859_2021_4238_MOESM2_ESM.docx]

**A B**

**
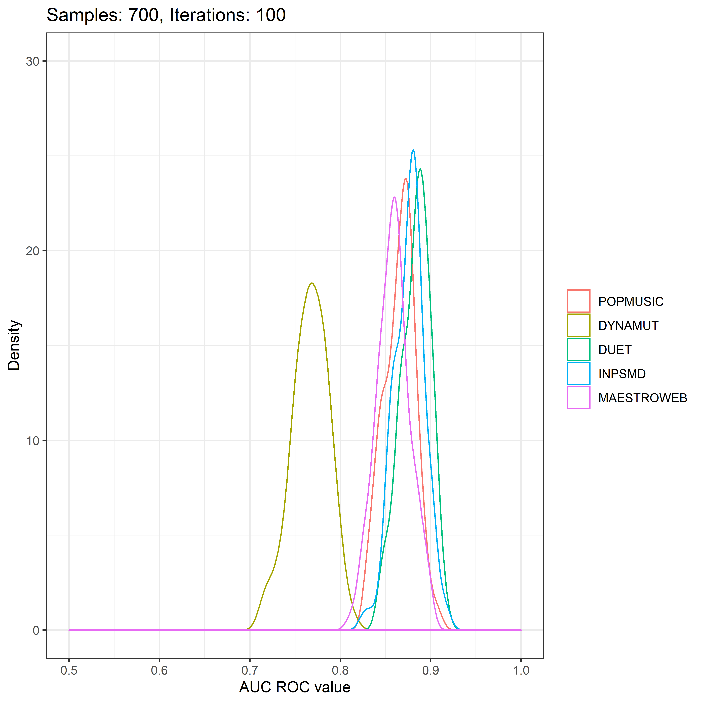

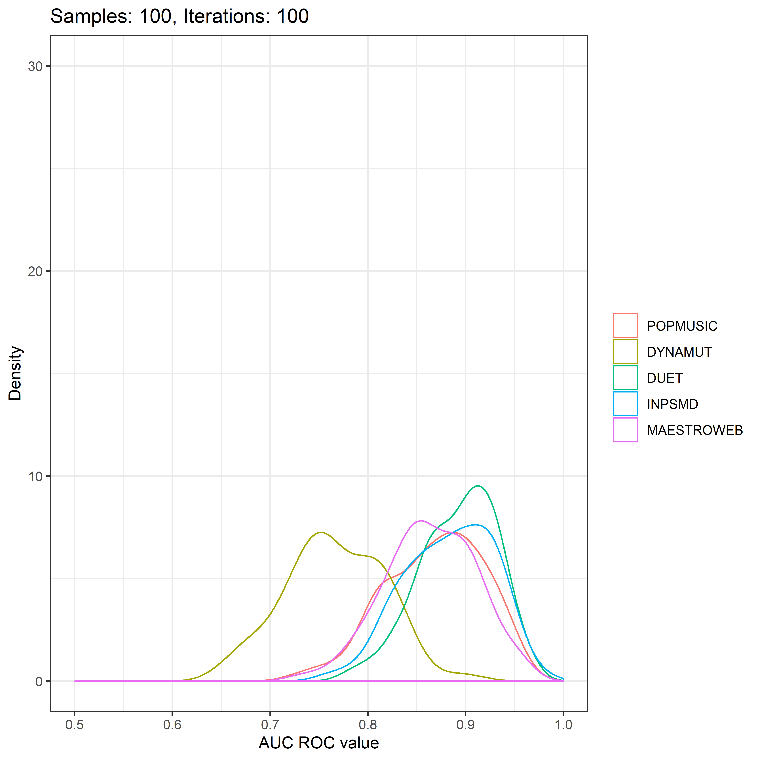
**

Supplement: Supplementary file 3 — Additional file 3: Fig. S3. AUC curve density obtained taking into account only those mutations outside the range of the experimental error, with a dataset of 100 (panel A) and 700 mutations (panel B), randomly extracted from the full dataset of monomeric proteins. [file 12859_2021_4238_MOESM3_ESM.docx]

**A B**


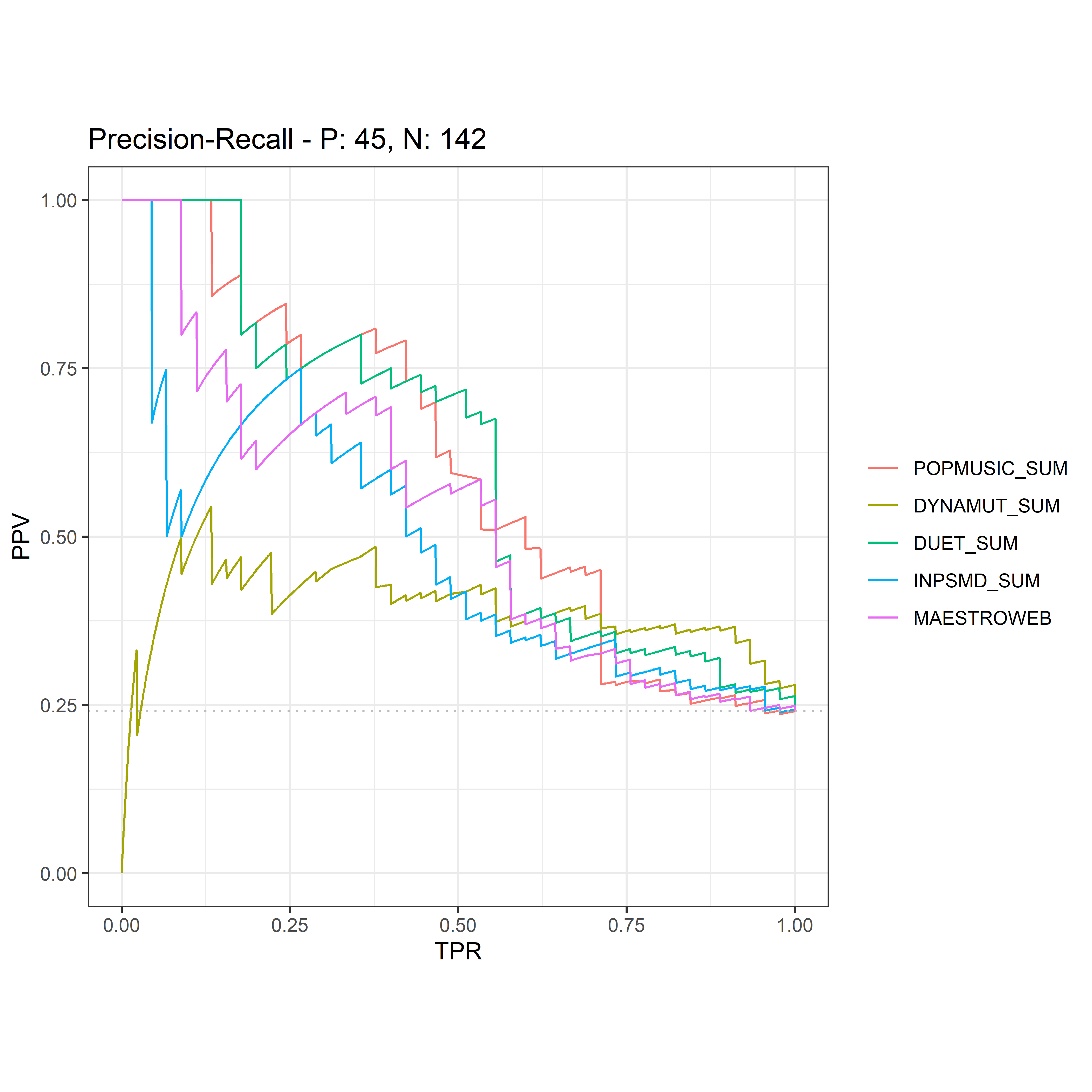

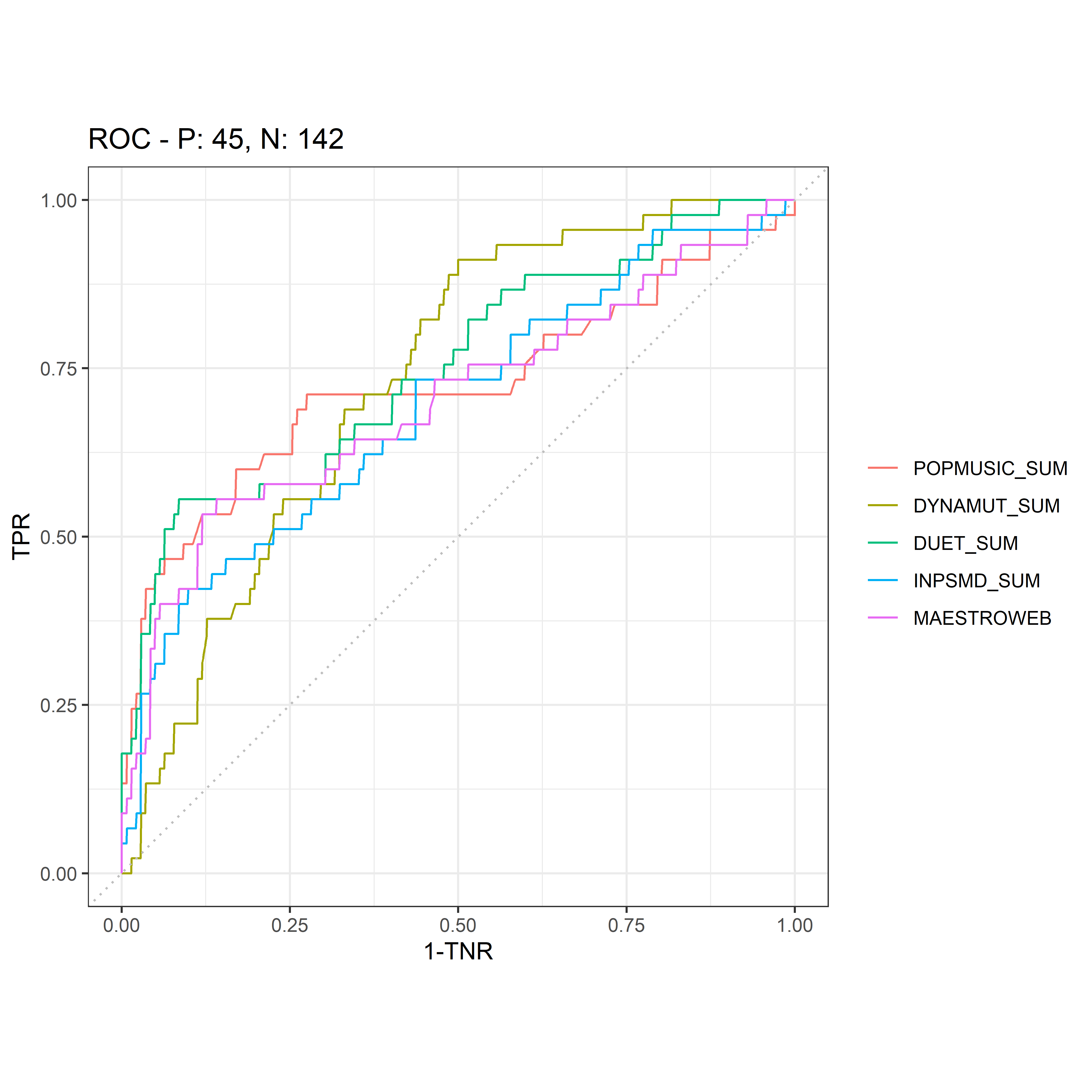

Supplement: Supplementary file 4 — Additional file 4: Fig. S4. ROC and PRC for predictions made on the full dataset of multimeric proteins. Panels (A) and (B) show the ROC and PRC, respectively, for predictions made on multimeric proteins, taking into account only those mutations outside the range of the experimental error (number of positive elements is 45, number of negative elements is 142). TNR: True Negative Rate, TPR: True Positive Rate, PPV: Positive Predictive Value. [file 12859_2021_4238_MOESM4_ESM.docx]

**A.**


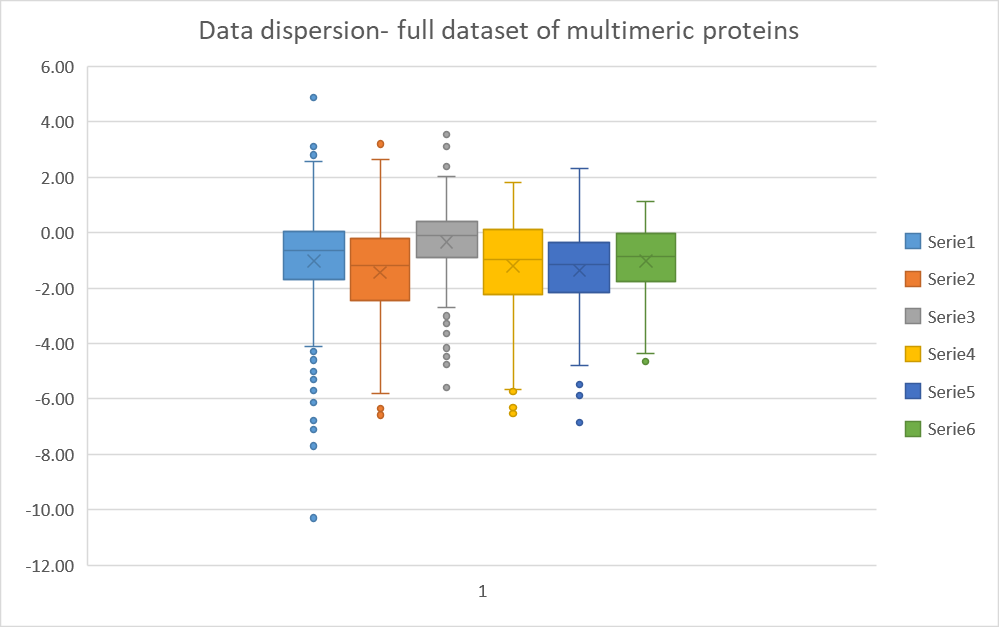


**B.**

**
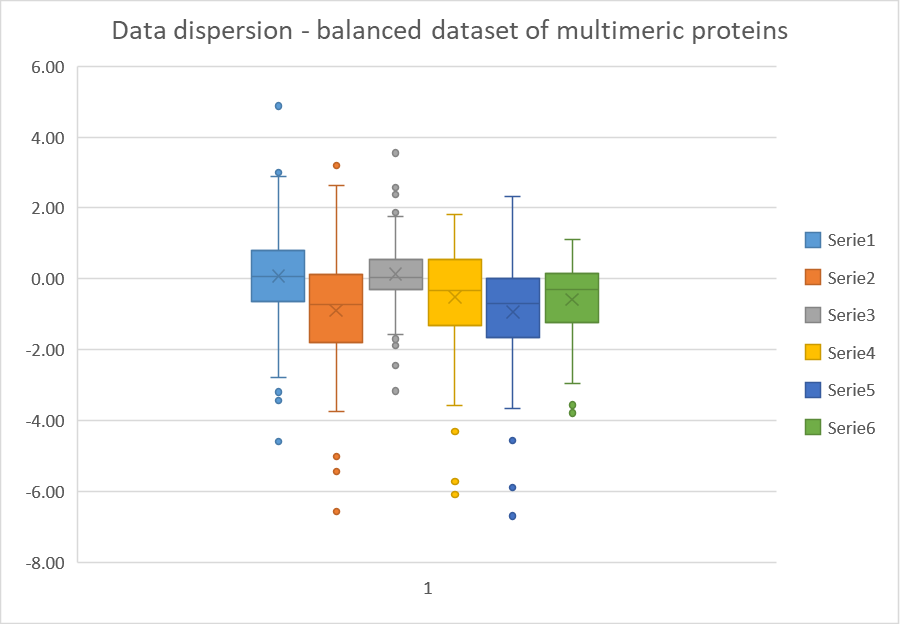
**

Supplement: Supplementary file 5 — Additional file 5: Fig. S5. Box-plot of the distribution of the experimental and predicted ΔΔG values for the full (panel A) and balanced (panel B) datasets of mutations for the multimeric proteins. Cyan: distribution of the experimental ΔΔG; orange: distribution of ΔΔG predictions for PoPMuSiC; grey: distribution of ΔΔG predictions for DynaMut; yellow: distribution of ΔΔG predictions for DUET; blue: distribution of ΔΔG predictions for INPS-MD; green: distribution of ΔΔG predictions for MAESTROweb. [file 12859_2021_4238_MOESM5_ESM.docx]

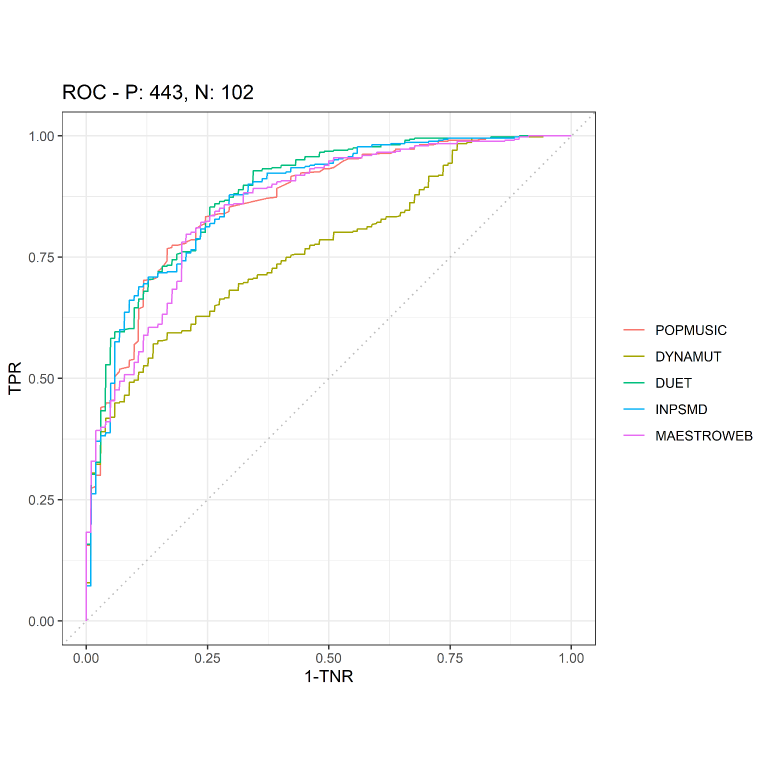

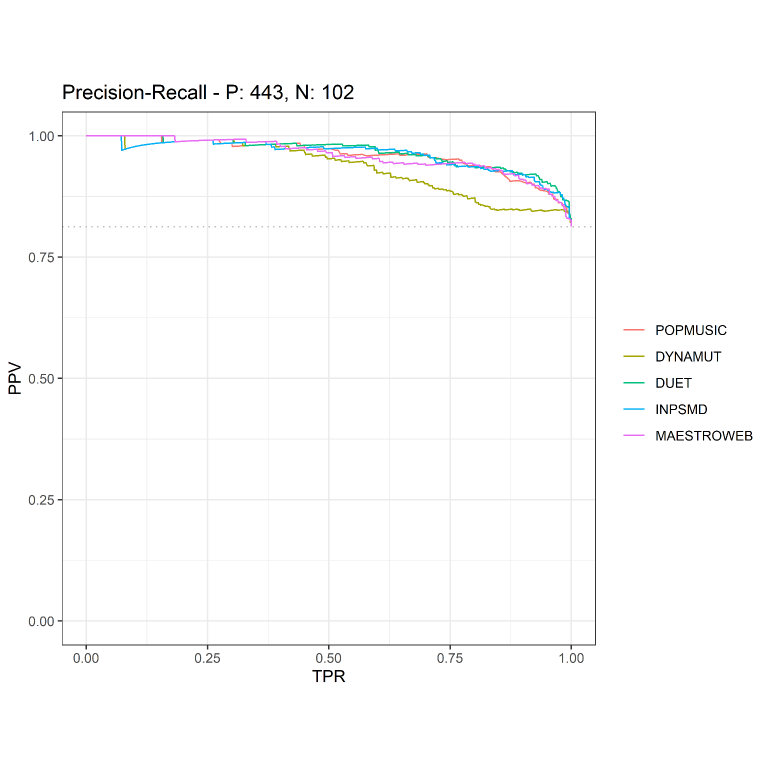

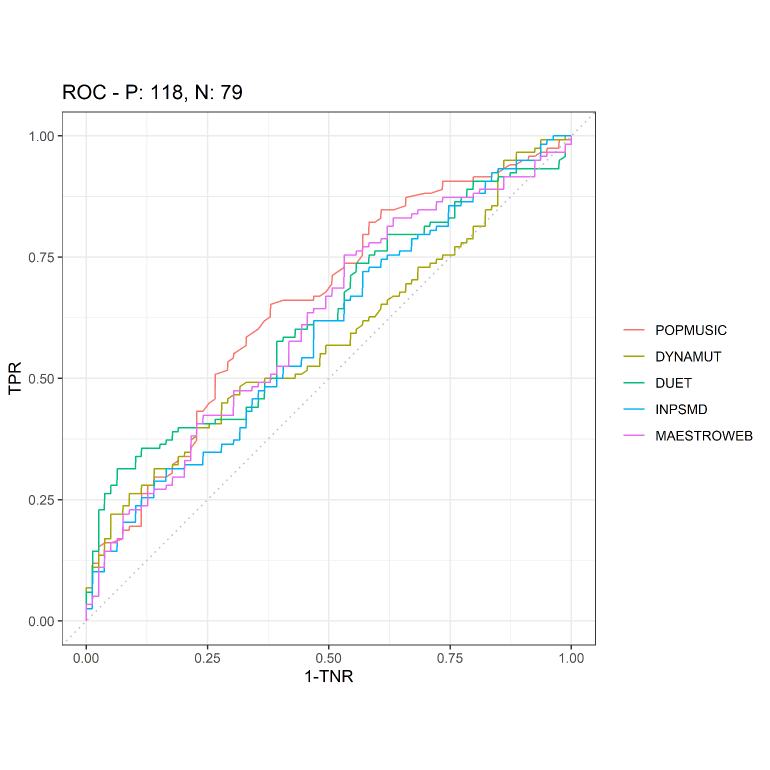

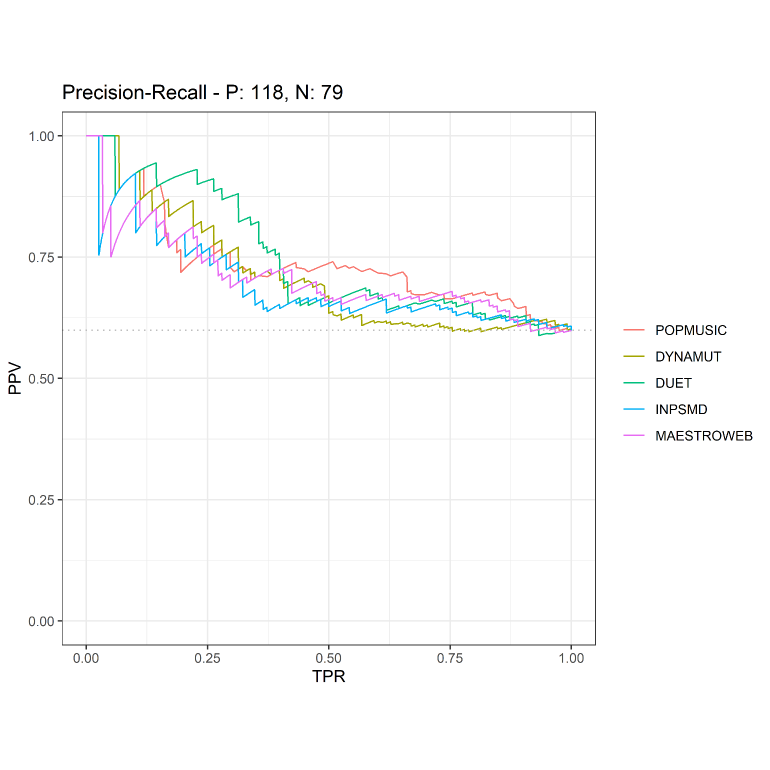


A B

C D

Supplement: Supplementary file 6 — Additional file 6: Fig. S6. ROC and PRC curves obtained as for Fig. 1 by considering negative ΔΔG predictions as positives. [file 12859_2021_4238_MOESM6_ESM.docx]

**A B**


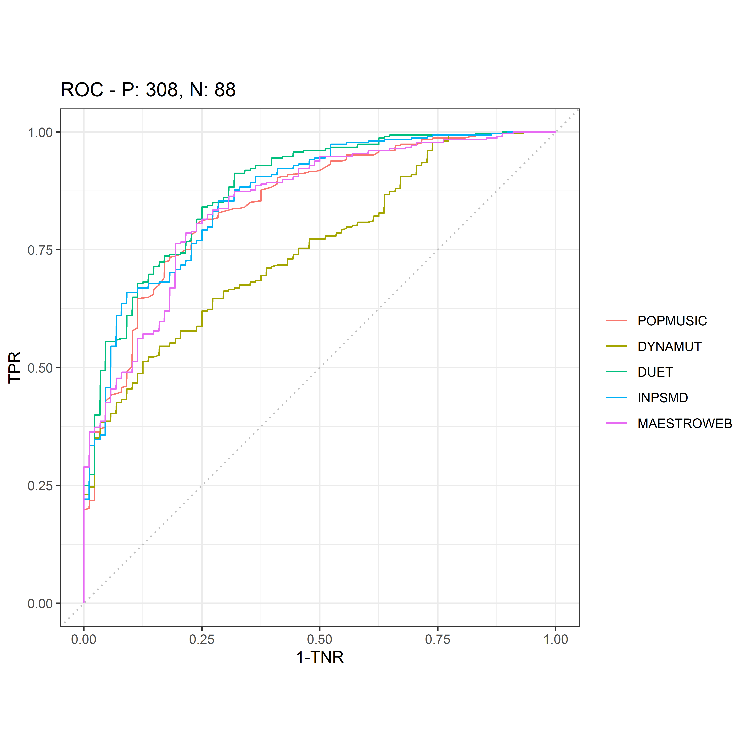


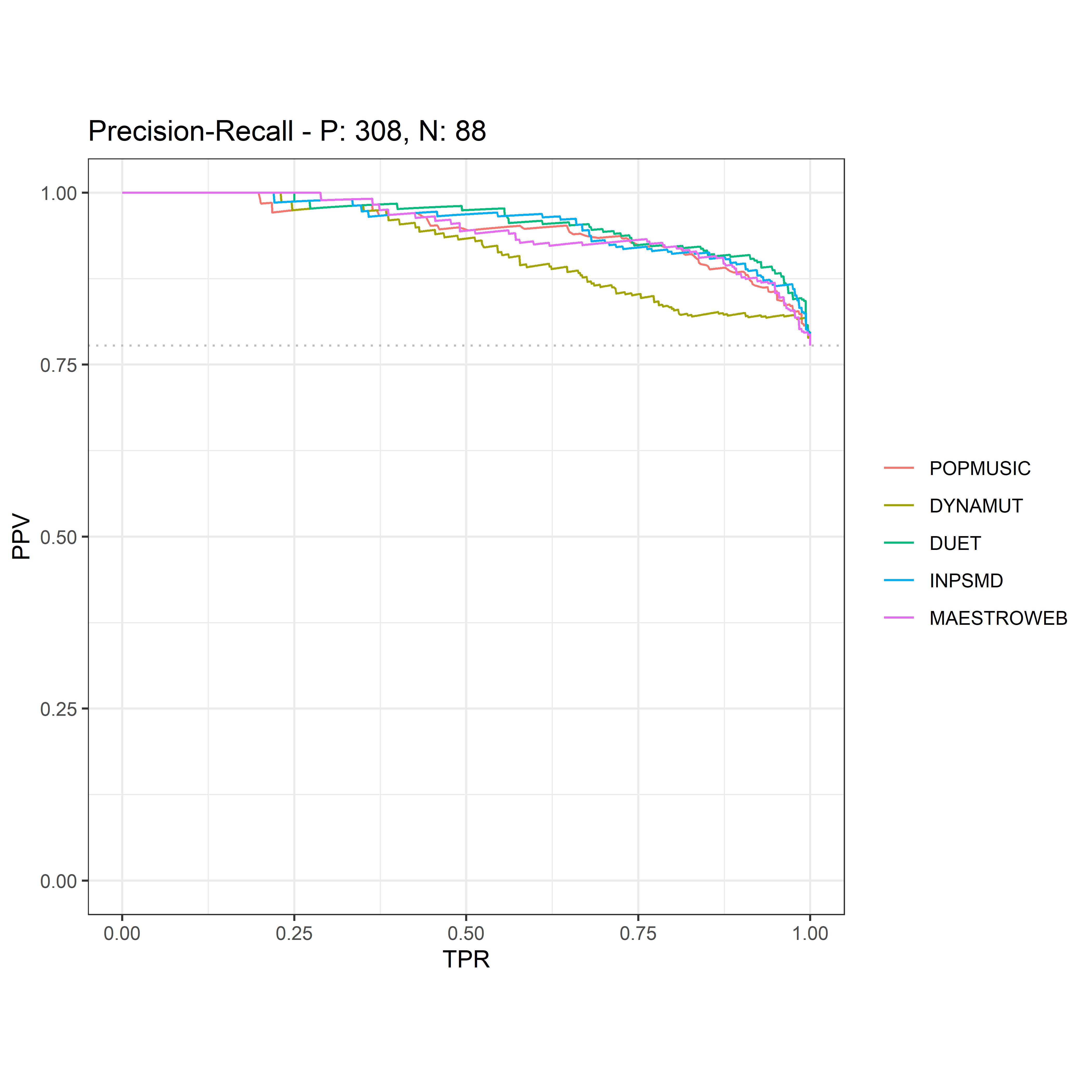

Supplement: Supplementary file 7 — Additional file 7: Fig. S7. ROC and PRC curves obtained as for Additional file 1: Figure S1 by considering negative ΔΔG predictions as positives. [file 12859_2021_4238_MOESM7_ESM.docx]

**A B**


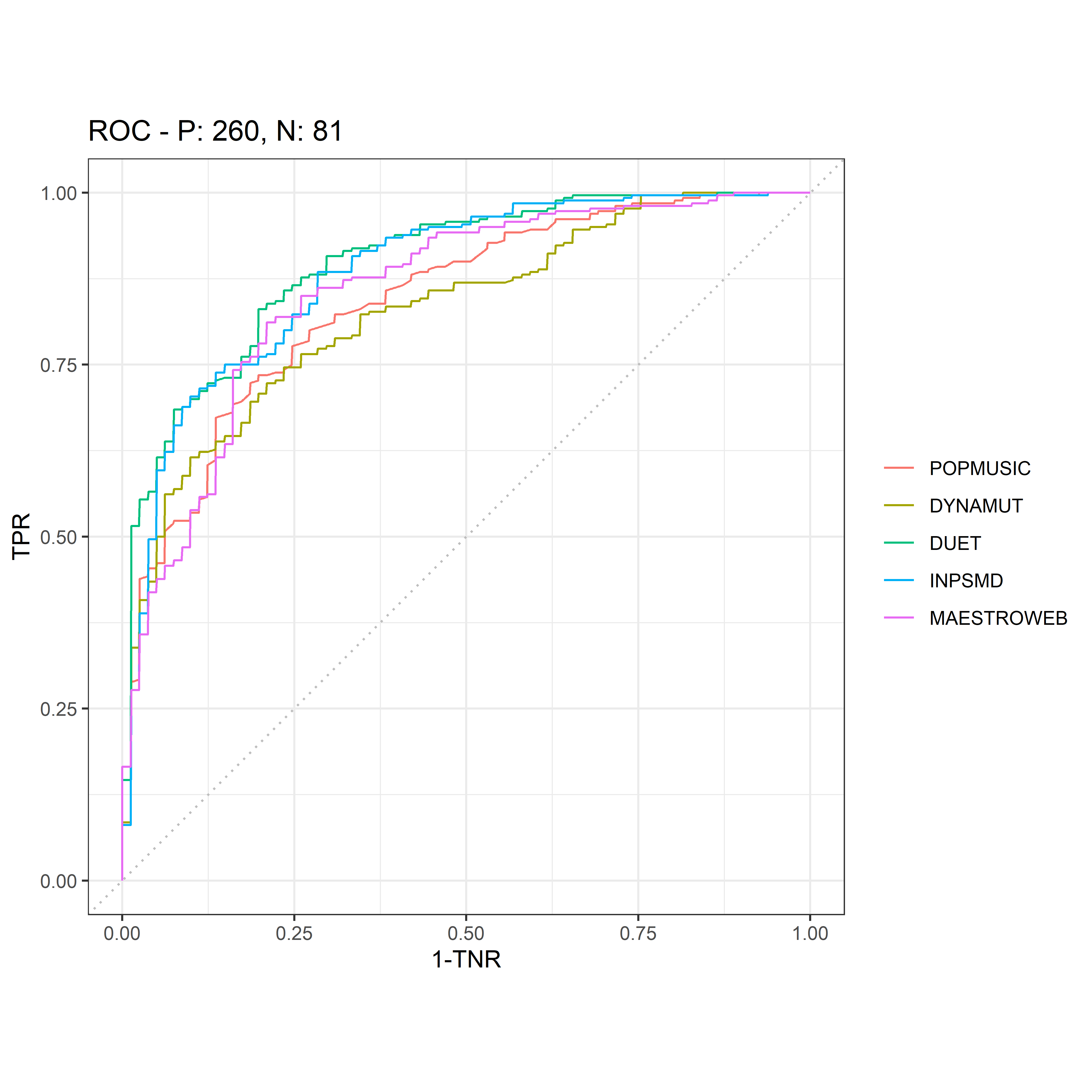

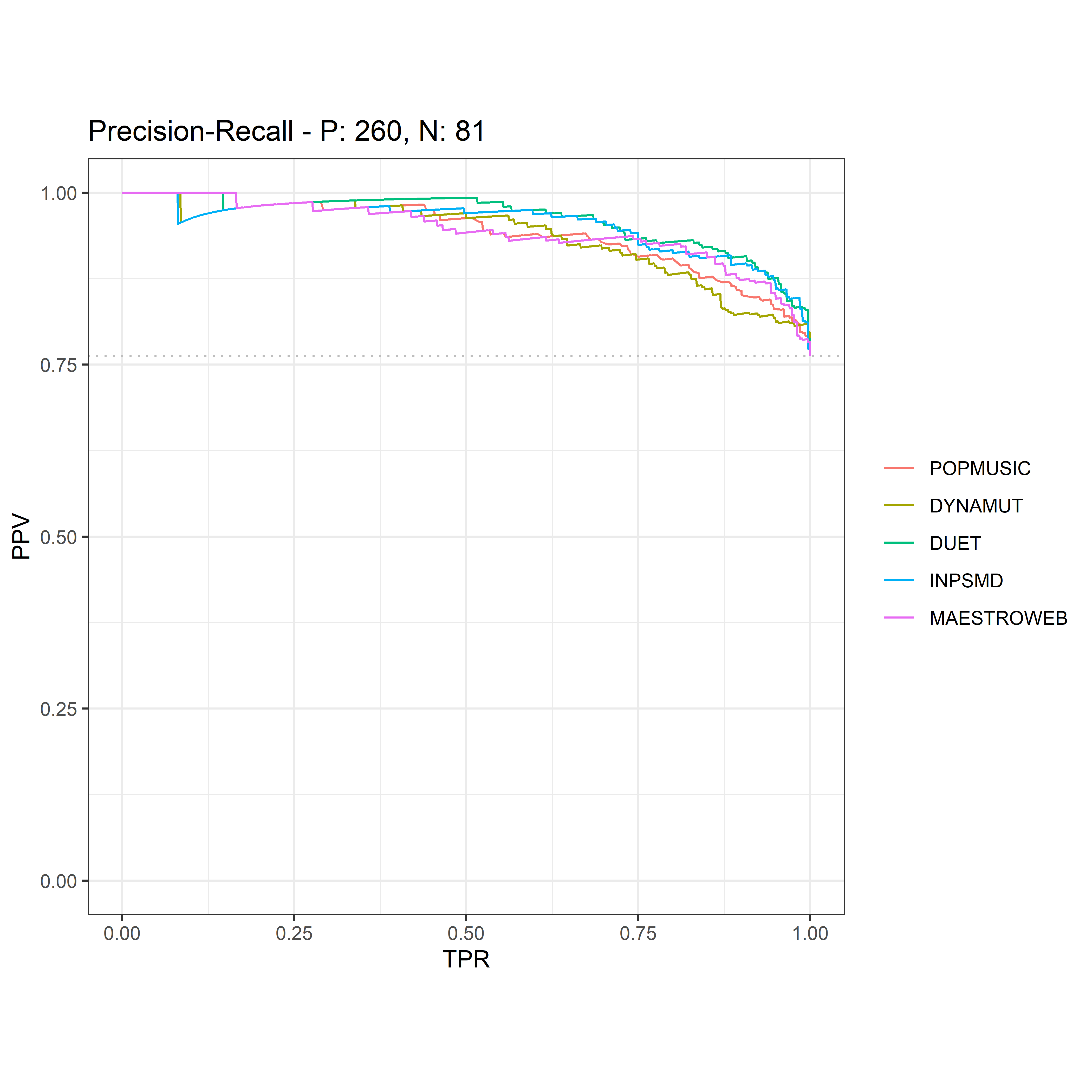

Supplement: Supplementary file 8 — Additional file 8: Fig. S8. ROC and PRC curves obtained as for Additional file 2: Figure S2 by considering negative ΔΔG predictions as positives. [file 12859_2021_4238_MOESM8_ESM.docx]

**A B**


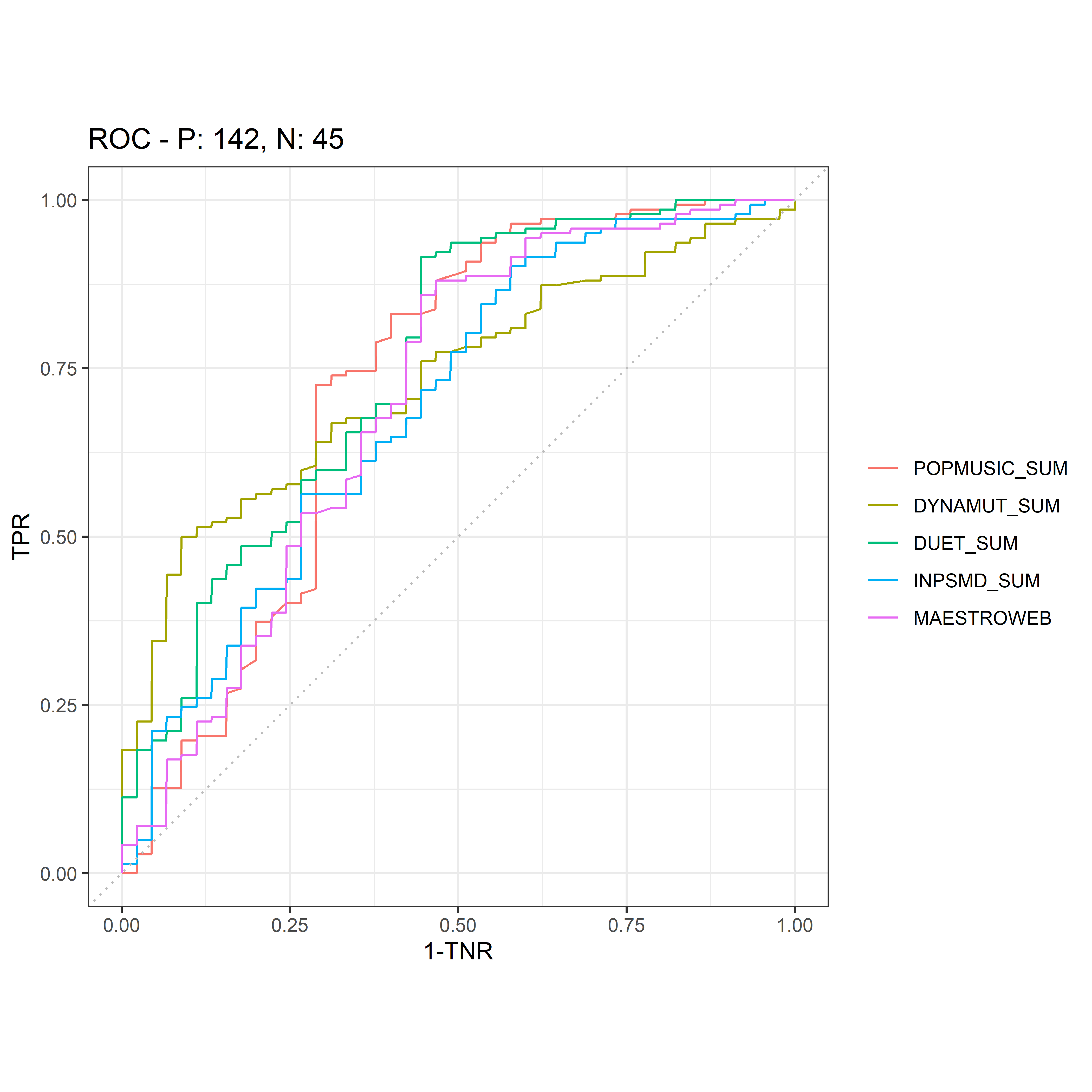

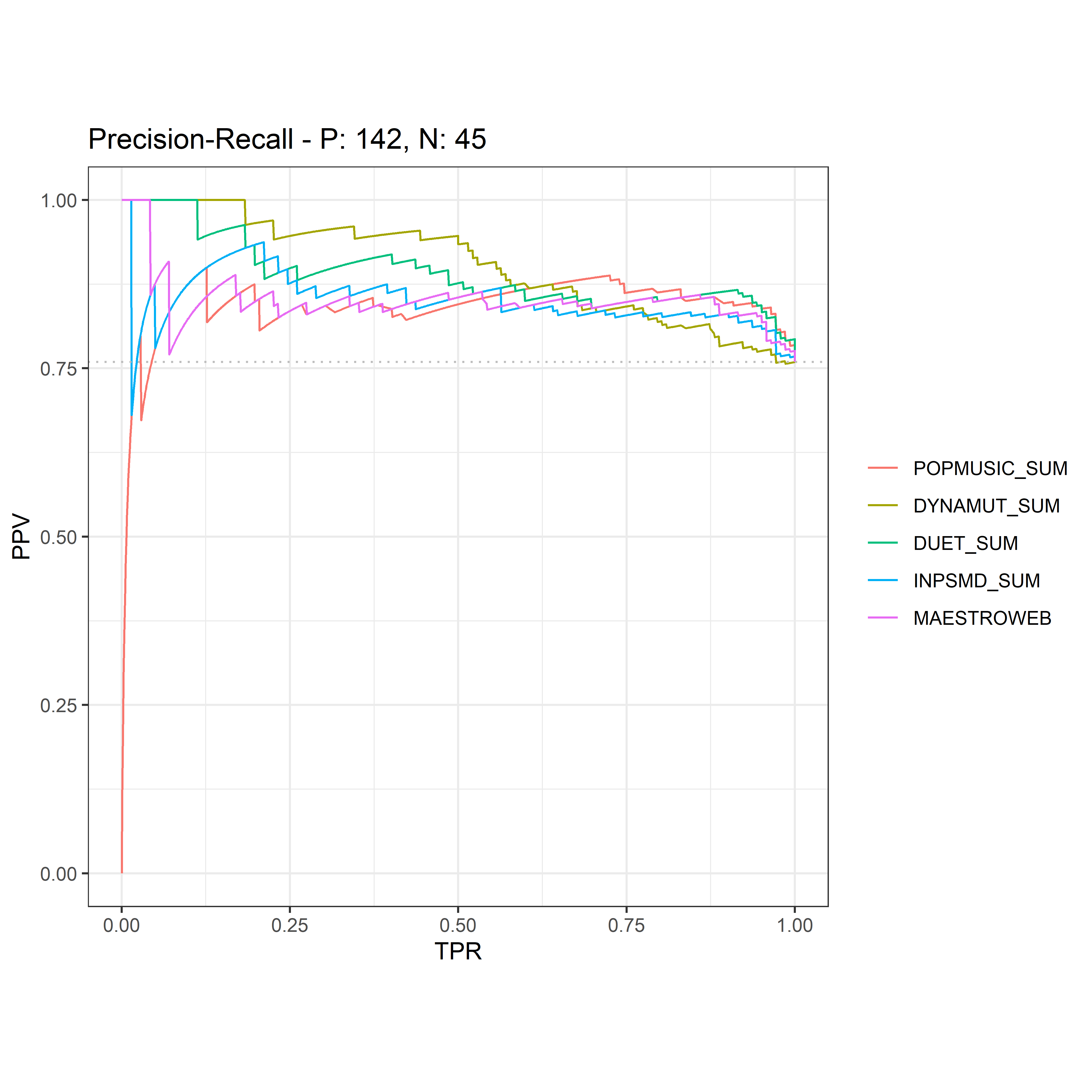

Supplement: Supplementary file 9 — Additional file 9: Fig. S9. ROC and PRC curves obtained as for Additional file 4: Figure S4 by considering negative ΔΔG predictions as positives. [file 12859_2021_4238_MOESM9_ESM.docx]
