# Supplementary material for: Performance of Web tools for predicting changes in protein stability caused by mutations
Source: BMC Bioinformatics. 2021 Jul 5;22(Suppl 7):345. doi: 10.1186/s12859-021-04238-w (PMC8256537; doi:10.1186/s12859-021-04238-w)
Supplement: Supplementary file 10 — Additional file 10: Table S1. Quantitative values of AUC calculated for the full dataset of monomeric structures. [file 12859_2021_4238_MOESM10_ESM.docx]

|  | **ROC OUT 0.5*** | **PRC OUT 0.5*** | **ROC IN 0.5**** | **PRC IN 0.5**** |
| --- | --- | --- | --- | --- |
| **PoPMuSiC** | 0,865 | 0,644 | 0,655 | 0,529 |
| **DynaMut** | 0,765 | 0,474 | 0,580 | 0,475 |
| **DUET** | 0,882 | 0,710 | 0,626 | 0,473 |
| **INPS-MD** | 0,874 | 0,681 | 0,593 | 0,499 |
| **MAESTROweb** | 0,859 | 0,633 | 0,617 | 0,480 |

* OUT 0.5: ΔΔG energy value outside the range [-0.5,+0.5] kcal/mol.

** IN 0.5: ΔΔG energy value inside the range [-0.5, +0.5] kcal/mol.
