# Supplementary material for: Performance of Web tools for predicting changes in protein stability caused by mutations
Source: BMC Bioinformatics. 2021 Jul 5;22(Suppl 7):345. doi: 10.1186/s12859-021-04238-w (PMC8256537; doi:10.1186/s12859-021-04238-w)
Supplement: Supplementary file 11 — Additional file 11: Table S2. Consensus among the sign of predictions made on monomeric proteins with more than one chain in their PDB file. [file 12859_2021_4238_MOESM11_ESM.docx]

|  | **PoPMuSiC*** | **DynaMut** | **DUET** | **INPS-MD** | **MAESTROWeb** |
| --- | --- | --- | --- | --- | --- |
| % of agreement among two chains | 100 | 91.9 | 95.2 | 100 | 96.8 |
| % of agreement among three chains | 100 | 73.6 | 93.9 | 97.8 | 95.0 |

*PoPMuSiC returns identical results (both in sign and in value) for all chains in a monomeric protein with multiple chains in its PDB file
