# Supplementary material for: Performance of Web tools for predicting changes in protein stability caused by mutations
Source: BMC Bioinformatics. 2021 Jul 5;22(Suppl 7):345. doi: 10.1186/s12859-021-04238-w (PMC8256537; doi:10.1186/s12859-021-04238-w)
Supplement: Supplementary file 12 — Additional file 12: Table S3. Consensus among the sign of predictions made on monomeric proteins among the different predictors. Values are expressed as percentage. [file 12859_2021_4238_MOESM12_ESM.docx]

|  | **PoPMuSiC** | **DynaMut** | **DUET** | **INPS-MD** | **MAESTROWeb** |
| --- | --- | --- | --- | --- | --- |
| **PoPMuSiC** | - | 58.76 | 82.74 | 83.53 | 82.74 |
| **DynaMut** | 58.76 | - | 66.53 | 59.29 | 62.19 |
| **DUET** | 82.74 | 66.53 | - | 80.36 | 81.15 |
| **INPS-MD** | 83.53 | 59.29 | 80.36 | - | 81.02 |
| **MAESTROWeb** | 82.74 | 62.19 | 81.15 | 81.02 | - |
