# Supplementary material for: Performance of Web tools for predicting changes in protein stability caused by mutations
Source: BMC Bioinformatics. 2021 Jul 5;22(Suppl 7):345. doi: 10.1186/s12859-021-04238-w (PMC8256537; doi:10.1186/s12859-021-04238-w)
Supplement: Supplementary file 14 — Additional file 14: File S2. Analysis of the composition of the full datasets [file 12859_2021_4238_MOESM14_ESM.docx]

Performance of Web tools for predicting changes in protein stability caused by mutations

Anna Marabotti^1^*, Eugenio Del Prete^2^, Bernardina Scafuri^1^, Angelo Facchiano^3^*

Supplementary Information

File 2

**Analysis of the composition of the full datasets**

**Composition of the high-quality reference dataset used for analysis**

**1) Selected proteins (those highlighted in yellow are in common with S2648 dataset)**

| **PDB code** | **Protein name** | **Biological assembly** | **Subunits in PDB file** | **CATH classification** | **Number of residues in PDB file** | **Number of mutations in database** | **Percentage of contribution to the database** |
| --- | --- | --- | --- | --- | --- | --- | --- |
| 1AAR | Ubiquitin from B. taurus | Dimer | A+B | 3.10.20.90 | 76 | 10 | 1.0 |
| 1AM7 | Lysozyme from lambda bacteriophage | Monomer | A, B, C | 1.10.530.10 | 158 | 2 | <1 |
| 1ANK | Adenylate kinase from E. coli | Monomer | A, B | 3.40.50.300 | 214 | 4 | <1 |
| 1AYF | Adrenodoxin bovine | Dimer | A+B | 3.10.20.30 | 105 | 11 | 1.1 |
| 1AZP | Sac7d from S. acidocalidarius | Monomer | A | 2.40.50.40 | 66 | 1 | <1 |
| 1BNI | Barnase from B. amyloliquefaciens | Monomer | A, B, C | 3.10.450.30 | 110 | 130 | 12.7 |
| 1BPI | Trypsin inhibitor bovine | Monomer | A | 4.10.410.10 | 58 | 42 | 4.1 |
| 1BVC | Sperm whale myoglobin | Monomer | A | 1.10.490.10 | 153 | 33 | 3.2 |
| 1C2R | Cytochrome C2 from R. capsulatus | Monomer | A, B | 1.10.760.10 | 116 | 5 | <1 |
| 1C5G | Plasminogen activator inhibitor 1 human | Monomer | A | 3.30.497.10 | 402 | 3 | <1 |
| 1C9O | Cold shock protein B (B. caldolyticus) | Dimer | A+B | 2.40.50.140 | 66 | 19 | 1.8 |
| 1CDC | Cell surface receptor protein CD2 from rat | Dimer | B+A | 2.60.40.10 | 99 | 9 | <1 |
| 1CHK | Chitosanase from Streptomyces | Monomer | A, B | 1.20.141.10 | 238 | 3 | <1 |
| 1CSP | Cold shock protein B from B. subtilis | Monomer | A | 2.40.50.140 | 67 | 40 | 3.9 |
| 1CYO | Cytochrome b5 bovine | Monomer | A | 3.10.120.10 | 93 | 10 | 1.0 |
| 1EL1 | Lysozyme (canine milk) | Monomer | A | 1.10.530.10 | 130 | 4 | <1 |
| 1FLV | Flavodoxin from Anaboena: oxidized long chain | Monomer | A | 3.40.50.360 | 169 | 2 | <1 |
| 1FTG | Apoflavodoxin from Anaboena | Monomer | A | 3.40.50.360 | 168 | 8 | <1 |
| 1G6N | Catabolite activator protein from E. coli | Dimer | A+B | 1.10.10.10 | 210 | 2 | <1 |
| 1HFY | Alpha-lactalbumin from C. hircus | Monomer | A, B | 1.10.530.10 | 123 | 3 | <1 |
| 1IGV | Calbindin D9k bovine | Monomer | A | 1.10.238.10 | 75 | 3 | <1 |
| 1IOB | Interleukin 1 beta human | Monomer | A | 2.80.10.50 | 153 | 7 | <1 |
| 1L63 | Lysozyme phage T4 (cysteine-free) | Monomer | A | 1.10.530.40 | 164 | 90 | 8.8 |
| 1LZ1 | Lysozyme human | Monomer | A | 1.10.530.10 | 130 | 6 | <1 |
| 1MGR | Ribonuclease Sa3 K. aureofaciens | Monomer | A | 3.10.450.30 | 99 | 8 | <1 |
| 1N0J | Superoxide dismutase [Mn] from rabbit | Tetramer | A+B+C+D | 1.10.287.990 | 199 | 3 | <1 |
| 1ONC | Onconase from L. pipiens | Monomer | A | 3.10.130.10 | 104 | 1 | <1 |
| 1PGA | Protein G from Streptococcus | Monomer | A | 3.10.20.10 | 56 | 33 | 3.2 |
| 1PIN | Pin1 WW domain human | Dimer | A | 3.10.50.40 | 163 | 56 | 5.5 |
| 1RGG | Ribonuclease Sa K. aureofaciens | Monomer | A, B | 3.10.450.30 | 96 | 45 | 4.4 |
| 1RN1 | Ribonuclease T1 A. oryzae | Monomer | A, B, C | 3.10.450.30 | 104 | 41 | 4.0 |
| 1ROP | Rop from E. coli | Dimer | A | 1.10.287.230 | 63 | 21 | 2.0 |
| 1RTB | Ribonuclease A bovine | Monomer | A | 3.10.130.10 | 124 | 49 | 4.8 |
| 1RTP | Alpha-parvalbumin R. norvegicus | Monomer | 1, 2, 3 (corrected into A, B, C) | 1.10.238.10 | 109 | 3 | <1 |
| 1RX4 | Dihydrofolate reductase from E. coli | Monomer | A | 3.40.430.10 | 159 | 12 | 1.2 |
| 1STN | Staphylococcal nuclease | Monomer | A | 2.40.50.90 | 149 | 39 | 3.8 |
| 1SUP | Subtilisin BPN' from B. amyloliquefaciens | Monomer | A | 3.40.50.20 | 275 | 6 | <1 |
| 1TPK | Plasminogen activator kringle-2 domain human | Monomer | A, B, C | 2.40.20.10 | 88 | 6 | <1 |
| 1VQB | Gene V from Enterobacteria phage F1 | Dimer | A | 2.40.50.140 | 87 | 92 | 9.0 |
| 1YCC | Iso-1 cytochrome c from S. cerevisiae | Dimer | A | 1.10.760.10 | 108 | 23 | 2.2 |
| 2AKY | Adenylate kinase S. cerevisiae | Monomer | A | 3.40.50.300 | 220 | 4 | <1 |
| 2DRI | Ribose-binding protein from E. coli | Monomer | A | 3.40.50.2300 | 271 | 2 | <1 |
| 2LZM | Lysozyme phage T4 (true wild type) | Monomer | A | 1.10.530.40 | 164 | 54 | 5.3 |
| 2RN2 | Ribonuclease HI from E. coli | Monomer | A | 3.30.420.10 | 155 | 40 | 3.9 |
| 2TRX | Thioredoxin from E. coli | Monomer | A, B | 3.40.30.10 | 108 | 2 | <1 |
| 3MBP | Maltose binding protein from E. coli | Monomer | A | 3.40.190.10 | 370 | 12 | 1.2 |
| 3SSI | Subtilisin inhibitor from S. albogriseolus | Dimer | A | 3.30.350.10 | 113 | 19 | 1.8 |
| 451C | Cytochrome C551 from P. aeruginosa | Monomer | A | 1.10.760.10 | 82 | 6 | <1 |

Total: 48 proteins, 1024 mutations.

**Distribution of mutations according to the experimental ΔΔG.**

|  | **Destabilizing**  **(ΔΔG< -0.5 kcal/mol)** | **Slightly destabilizing, more uncertain**  **(-0.5≤ΔΔG<0 kcal/mol)** | **No effect**  **(ΔΔG=0 kcal/mol)** | **Slightly stabilizing, more uncertain**  **(0<ΔΔG≤0.5 kcal/mol)** | **Stabilizing**  **(ΔΔG>0.5 kcal/mol)** | **Total** |
| --- | --- | --- | --- | --- | --- | --- |
| **Monomeric proteins, full dataset** | 443 | 118 | 17 | 79 | 102 | 759 |
| ***Monomeric proteins, balanced dataset*** | *102* | *80* | *0* | *79* | *102* | *363* |
| **Multimeric proteins, full dataset** | 142 | 50 | 4 | 24 | 45 | 265 |
| ***Multimeric proteins, balanced dataset*** | *38* | *22* | *0* | *24* | *45* | *129* |
| **General dataset** | 585 | 168 | 21 | 103 | 147 | 1024 |

**Proteins contributing ≥5% of the mutations of the dataset:**

Lysozyme (5 proteins, 156 mutations, 15.2% of the dataset)

Ribonuclease (5 proteins, 183 mutations, 17.9% of the dataset)

Cold shock protein (2 proteins, 59 mutations, 5.8% of the dataset)

**Protein length:**

15 proteins with <100 residues (31.2%)

25 proteins with ≥100 and ≤200 (52.1%)

8 proteins with >200 residues (16.7%)

**Protein classification**

1: Mainly alpha: 16 (33.3%)

2: Mainly beta: 8 (16.7%)

3: Alpha Beta: 23 (47.9%)

4: Few secondary structures: 1 (2.1%)

**2) Distribution of the mutations in the entire dataset**

a) Mutations from the original wild type residue to any residue

b) Mutations from any residue to the mutant residue:

**3) Distribution of the mutations in the monomer dataset**

a) Mutations from the original wild type residue to any residue

b) Mutations from any residue to the mutant residue:

**3) Distribution of the mutations in the multimer dataset**

a) Mutations from the original wild type residue to any residue

b) Mutations from any residue to the mutant residue:

**Composition of the datasets for proteins contributing ≥5% of the dataset**

**1BNI - Barnase from B. amyloliquefaciens**

a) Distribution of mutations along the sequence (numbering according to PDB file)

b) Distribution of mutations according to the type of original residue

c) Distribution of mutations according to the type of mutant residue

**2) 1L63 - Lysozyme from phage T4 “cysteine-free”**

a) Distribution of mutations along the sequence (numbering according to PDB file)

b) Distribution of mutations according to the type of original residue

c) Distribution of mutations according to the type of mutant residue

**3) 1PIN - PIN1 WW domain human**

a) Distribution of mutations along the sequence (numbering according to PDB file)

b) Distribution of mutations according to the type of original residue

c) Distribution of mutations according to the type of mutant residue

**4) 1VQB - Gene V from Enterobacteria phage F1**

a) Distribution of mutations along the sequence (numbering according to PDB file)

b) Distribution of mutations according to the type of original residue

c) Distribution of mutations according to the type of mutant residue

**2LZM - Lysozyme from phage T4 (true wild type)**

a) Distribution of mutations along the sequence (numbering according to PDB file)

b) Distribution of mutations according to the type of original residue

c) Distribution of mutations according to the type of mutant residue
